# The effect of blood sampling and preanalytical processing on human N-glycome

**DOI:** 10.1371/journal.pone.0200507

**Published:** 2018-07-11

**Authors:** Tereza Dědová, Detlef Grunow, Kai Kappert, Dagmar Flach, Rudolf Tauber, Véronique Blanchard

**Affiliations:** 1 Charité –Universitätsmedizin Berlin, Institute of Laboratory Medicine, Clinical Chemistry and Pathobiochemistry, Berlin, Germany; 2 Freie Universität Berlin, Department of Biology, Chemistry and Pharmacy, Berlin, Germany; 3 Center for Cardiovascular Research, German Center for Cardiovascular Research, Charité –Universitätsmedizin Berlin, Berlin, Germany; 4 Sarstedt AG&Co, Nümbrecht, North Rhine-Westphalia, Germany; Edith Cowan University, AUSTRALIA

## Abstract

Glycome modulations have been described in the onset and progression of many diseases. Thus, many studies have proposed glycans from blood glycoproteins as disease markers. Astonishingly, little effort has been given unraveling preanalytical conditions potentially influencing glycan analysis prior to blood biomarker studies. In this work, we evaluate for the first time the effect of hemolysis, storage and blood collection, but also influence of various times and temperatures between individual processing steps on the total N-glycome and on a glycan-biomarker score. Venous blood was collected from 10 healthy donors in 11 blood collection tubes with different additives, processed variously to obtain 16 preanalytical variables and N-glycans released from serum or plasma were analyzed by MALDI-TOF-MS and capillary electrophoresis coupled with fluorescence detection (CE-LIF) for the first time. Long time storage of deep frozen samples at -20°C or -80°C exerted only a minor influence on the glycome as demonstrated by CE-LIF. The N-glycome was very stable evidenced by MALDI-TOF when stored at 4°C for at least 48 hours and blood collected in tubes devoid of additives. The glycome was stable upon storage after centrifugation and aliquoting, which is an important information considering future diagnostic applications. Hemolysis, however, negatively correlated with an established glycan score for ovarian cancer, when evaluated by MALDI-TOF-MS measurement by affecting relative intensities of certain glycans, which could lead to false negative / positive results in glycan biomarker studies.

## Introduction

With the exception of albumin, most abundant serum and plasma proteins are co- and post-translationally glycosylated with N- and O-glycans [1]. Protein glycosylation effects various biological roles such as prolongation of protein half-life due to reduced clearance and protection from proteolysis [2, 3]. In addition, it also mediates the interaction of proteins with glycan-specific receptors in the context of a wide variety of cell functions, for instance cell-cell communication, signal transduction or immune response [3–6]. The glycans of serum and plasma glycoproteins are referred to as the serum and plasma glycome. In recent years, research has shown that not only the monosaccharide composition, but also the structure of the N- and O-glycans of serum and plasma glycoproteins is modulated under pathological conditions. This includes genetic diseases [7], acute and chronic inflammation [8–10] and malignant diseases such as colon and ovarian cancer [11–13]. In the past decades, glycan analysis has been hampered by time-consuming analytical procedures. Recently, methods for high-throughput analysis have been developed that allow the investigation of large numbers of samples [11, 14, 15]. A growing number of studies established that changes in glycome are not only of pathogenic importance, but can be used as diagnostic biomarkers. In particular, glycome variations are a hallmark of malignancy with glycome modulations being cancer-specific or -associated [16–21]. Such a glycan-based cancer biomarker, the GLYCOV index score, developed in our laboratory, combined qualitative and quantitative changes of serum glycome in patients suffering from epithelial ovarian cancer [11, 12]. Its sensitivity and specificity were compared with that of the established tumor marker CA125 and indicated superior accuracy, even for early-stage patients.

The analytical methods used to study the glycome are very well established; MALDI-TOF-MS, capillary electrophoresis coupled with fluorescence detection (CE-LIF) and HPLC being among the most used technologies [15, 22]. Intra-individual stability of the glycome in time has been well studied, demonstrating the influence of the lifestyle, medication or environmental factors [23, 24]. It was reported by Ventham *et al*.[25] that, when a standard processing methodology is used, variables such as tube manufacturer, tube volume and addition of separation gel as well as centrifugation time, temperature and speed, have little effect on results of HPLC measurements of human serum N-glycome. Another report by Adamczyk *et al*.[26] described that N-glycome differences between serum and plasma are caused mainly by fibrinogen N-glycosylation and that the presence or absence of anticoagulant does influence results of HPLC glycan analysis regardless of anticoagulant type. Although there have been about 15,000 entries in PubMed for glycan biomarkers in the past ten years, the preanalytical conditions of MALDI-TOF-MS and CE-LIF glycome analysis have never been reported in detail. However, preanalytical variables may impact significantly glycome profiles and can lead to inaccurate results as reported for peptide profiling [27–29]. It can be assumed that the activity of exoglycosidases and glycosyltransferases that are detectable in serum such as sialidase, mannosidase and sialyltransferase, will depend on the temperature of the sample and the time until storage and/or measurement. As for proteomics [27, 28], the influence of sample freezing could also lead to glycan degradation. Moreover, glycoproteins may be released during the preanalytical phase from intracellular organelles or by shedding of the cell surface of leukocytes and erythrocytes, which would result in changes of the serum glycome. With the increased popularity of international and multi-center studies [22, 30–32], it becomes necessary to evaluate the effect of pre-analytical conditions that are more difficult to control and standardise, such as time periods between individual processing steps, which depend greatly on the method of transportation. For example, transportation from collection site to processing laboratory can greatly differ. On-site laboratories have the advantage of complete control over preanalytical process, while courier pick-up and pneumatic tube system introduce variables such as delivery times, temperatures and physical damage. In general, higher occurrence of hemolysis was reported for samples transported by pneumatic tube system, but this differs from system to system [33, 34]. To our knowledge, there is no research published on the effects of hemolysis on the results of N-glycome analysis, namely individual glycans and glycan scores. In this work, we evaluate for the first time not only the effect of hemolysis, storage and blood collection, but also influence of various times and temperatures between individual processing steps on the N-glycome using blood samples from healthy volunteers and detection with MALDI-TOF-MS and CE-LIF methods. Additionally, we study the influence of preanalytical conditions not only on single glycan structures but also on a glycan-based ovarian cancer biomarker GLYCOV [11], which is an index score built from the relative intensities of eleven glycan structures.

## Materials and methods

### Blood sampling procedure

Blood from 10 healthy female donors (20–30 years) was collected according to the ethical approval of the Charité Medical University EA1/175/14 in eleven different collection tubes per donor. All collection tubes were S-Monovette tubes (Sarstedt, Germany) (Table 1). Plasma samples were collected in five tubes containing either K3 EDTA (n = 1, Tube I), trisodium citrate solution (n = 3, Tubes G, H, K) or heparin (n = 1, Tube J). Six serum samples were collected using 5 tubes containing clotting activating silica-covered beads without separation gel (Tubes A-D) and one tube with separation gel (Tube E). To evaluate the influence of the collection method, one evacuated blood collection tube was used for collection of citrated plasma sample (Tube G), the 10 other tubes were collected by free flow. The blood collections were performed in two sets of five donors six months apart. The second collection was performed after initial data analysis and one additional condition was tested.

**Table 1:** List of serum and plasma collection tubes used in this study.

Tube Nr.	Tube	Tube material	Separator	Clot activator	Anticoagulant
A-D	S-Monovette® 4.9ml Z	Polypropylene (PP)	None	Silicate coated beads	None
E	S-Monovette® 4ml Z-Gel	Polypropylene (PP)	Polyacrylic ester gel	Silicate coated beads	None
F	S-Monovette® 9ml Z	Polypropylene (PP)	None	Silicate coated beads	None
G-H, K	S-Monovette® 5ml 9NC	Polypropylene (PP)	None	None	Trisodium citrate solution 3.2% (1:10)
I	S-Monovette® 4.9ml K3E	Polypropylene (PP)	None	None	K3 EDTA
J	S-Monovette® 4.9ml LH	Polypropylene (PP)	None	None	Lithium heparin

### Blood preanalytical processing

**Fig 1:**
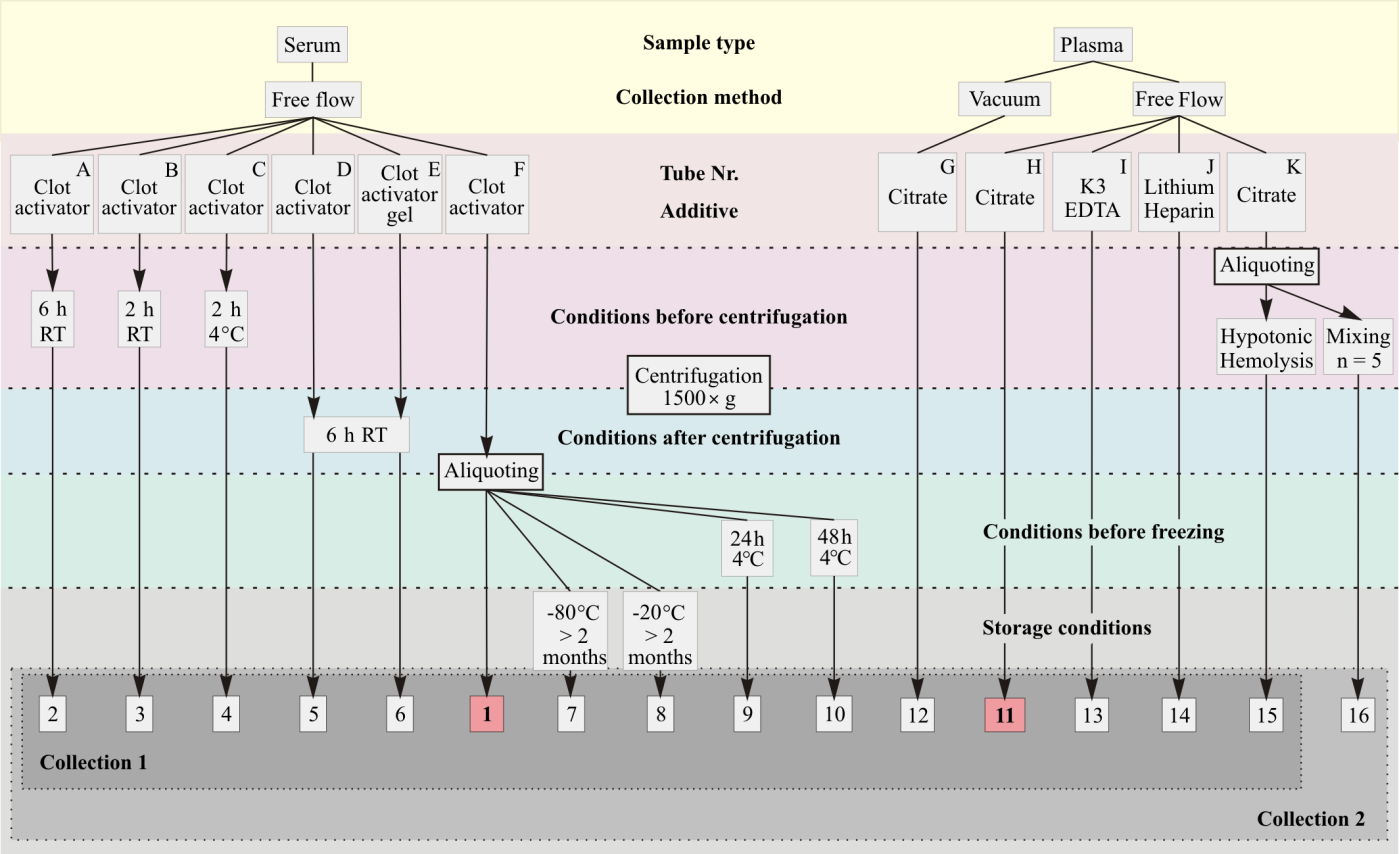
Flowchart showing the preanalytical processing carried out in this study. Reference conditions (time frames between processing steps as short as possible) are marked in red. Samples were kept for 30 min at RT, were subsequently centrifuged for 15 minutes at 1500×g and 4°C, aliquoted immediately after centrifugation and stored at -80°C, unless stated otherwise. Conditions 1–15 were created for the first set of collection, conditions 1–16 were created for the second set of collection. It should be noted that tubes F and K were used for generating multiple conditions.

Samples were kept in an up-right position except during centrifugation. Tubes were further processed to generate a total of 16 different conditions (Fig 1). Reference plasma and serum samples were collected in a trisodium citrate tube (tube H) and a serum clot activator tube (tube F), respectively. To evaluate the influence of delayed centrifugation, three collection tubes were kept at room temperature (RT) for 2 h and 6 h, or at 4°C for 2 h (tubes A, B and C, respectively). Seven tubes (tubes D-J) were left for 30 min at RT and were then centrifuged at 1500×g at 4°C for 15 min. Collection tubes D and E (clotting activator and clotting activator with separation gel) were left at RT for 6 h after centrifugation. The last tube (tube K) was used to induce two hemolytic conditions before centrifugation. Additionally, for the second collection set (n = 5), one milliliter of citrated whole blood from the same tube was aliquoted and shaken in 1.5 ml polypropylene tubes placed in an up-right position on a Thermomixer comfort (Eppendorf, Hamburg, Germany) at maximum speed of 1400 rpm overnight at RT to test extreme deviations from standard preanalytical procedures. The remaining citrated blood was hemolysed directly in the collection tube using hypotonic conditions by addition of deionized water (MilliQ, Millipore) for all ten donors in both sets. Hemolysis was confirmed visually for both samples hemolysed by hypotonic conditions (n = 10) and by mixing (n = 5), and free hemoglobin was measured to quantify the degree of hemolysis. Two hundred microliters of each sample were then aliquoted and stored at -80°C or -20°C.

### N-Glycan release and purification

All samples were subjected to glycomic profiling as described in a previous study by Biskup et al. without any modification [11]. Briefly, N-glycans were released and isolated from 10 μl of sample, *e*.*g*., the serum/ plasma was diluted in phosphate buffer (pH 6.8), reduced with dithioerythritol (DTE; Sigma-Aldrich) and alkylated with iodoacetamide (IAA; Sigma-Aldrich). Samples were prepared in triplicate. Excess of DTE was used to stop the reaction and 100 mU of Peptide-N-glycosidase F (PNGase F; EC 3.5.1.52; Roche Applied Science, Indianapolis, IN) were used for N-glycan release at 37°C overnight. Samples were purified using C18 cartridges and desalting was performed by graphitized carbon columns (both purchased from Alltech, Deerfield, IL).

### Mass spectrometry

Permethylation was performed as published by Wedepohl et al. [35]. After termination of the reaction, permethylated glycans, purified by chloroform extraction, were washed with water until the pH of the aqueous phase was neutral. Chloroform was removed under reduced atmosphere, the sample dissolved in 5 μl 75% aqueous acetonitrile and 0.5 μl of the sample was mixed with 0.5 μl 10 mg/ml super DHB matrix (2-hydroxy-5-methoxy-benzoic acid and 2,5-dihydroxybenzoic acid, 1:9; Sigma-Aldrich), prepared in 10% acetonitrile, 90% water, on a ground steel MALDI target (Bruker Daltonics, Bremen, Germany) and used for MALDI-TOF-MS measurement. Spectra were recorded in a reflectron positive mode on an Ultraflex III mass spectrometer (Bruker Daltonics, Bremen, Germany) equipped with a Smartbeam laser. A glucose ladder was used for calibration and 2000 laser shots were recorded for each spectrum in the mass range 1000–5000 Da with ion suppression below 990 Da (100 Hz laser frequency). The accelerating voltage was 25kV. Based on the knowledge of N-glycan biosynthetic pathways and specificity of PNGase F, all N-glycans were presumed to have N2H3 core structure and the compositions (numbers of H, N, F, S) were manually interpreted based on their *m/z* values. MALDI-TOF-MS spectra were annotated manually with the assistance of GlycoWorkBench (version 1.1.3480) glycoinformatic tool and literature [1] and exported using the Flexanalysis Software (Version 3.0, Build 54, Bruker Daltonics, Bremen, Germany) as ASCII files. The resulting peak lists were used for targeted data extraction of the area under the curve after manual recalibration using glycan peaks with known compositions (H3N4F1, H4N4F1, H5N4F1, H5N4S1, H5N5F1S1, H5N4S2, H6N5S3, H6N5F1S3 and H7N6S4), baseline detection and subsequent subtraction from intensities of all isotopic peaks using Python Script published by Reiding et al. [36] with permethylated masses as building blocks (S1 Table), sodium as charge carrier and a calculation window of 0.49 *m/z*. Complete MALDI-TOF-MS data are presented in S1 File.

### Capillary electrophoresis

Samples were chemically desialylated with 0.5 M acetic acid at 80°C for 3 h and 8-aminopyrene-1,3,6-trisulfonic acid (APTS) derivatization was performed as described earlier **[37]**. A Beckman P/ACE MDQ system equipped with LIF detection (λ_ex_ = 488 nm, λ_em_ = 510 ± 10 nm) (Beckman Coulter, Fullerton, CA, USA) was used to perform CE separations of APTS-labeled N-glycans by reversed polarity on a polyvinyl alcohol-coated capillary (Beckman, 50 μm I.D., total capillary length and effective separation length from the injection to the detector were 65 cm and 50 cm, respectively). Samples were injected for 10 s at a pressure of 0.5 psi and separations were performed using an applied potential of 30 kV for 20 min at 25°C. APTS-labeled maltose was used as an internal standard. Signals from CE-LIF measurements, exported as ASCII files, were aligned using a Python script HPACED [38] based on the highest peak of the spectrum. The software OpenChrom (Community edition 1.1.0) was used to perform baseline subtraction, signal smoothing and signal integration, which was automatically performed using the Peak Detector integration function. Complete CE-LIF data are presented in S1 File.

### Statistical analysis

SPSS for Windows, version 21 (SPSS Inc, Chicago, Ill) was used for statistical analysis. All samples were prepared in replicates (n = 3 for MALDI-TOF-MS, n = 2 for CE-LIF) and their means were calculated. Shapiro-Wilk normality test showed non-normal distribution of some glycans, therefore non-parametric tests were used to assess differences between conditions. Friedman’s non-parametric test was used on the complete data set (all conditions from 10 persons) to test if there were differences in relative intensities of individual glycans between different preanalytical conditions and p values < .05 were considered statistically significant. Post-hoc analysis with Wilcoxon signed-rank tests was performed with a Bonferroni correction applied, therefore significance levels were set at p < .0056 (n of comparisons = 9) for serum conditions and p < .01 (n of comparisons = 5) for plasma conditions.

## Results

### N-Glycan profiles obtained with standard conditions

The total serum and plasma N-glycome profiles of 10 healthy women with age 20–30 years, collected in various blood collection tubes and processed to obtain 16 different conditions, were analysed in triplicates, leading to 465 samples total, of these 60 were obtained from reference serum and plasma test tubes. Reference conditions were set in such a manner that the time frames between processing steps such as centrifugation and aliquoting were as short as possible, namely 30 min between blood collection and centrifugation, followed by immediate aliquoting and storage. N-Glycans were released by PNGase F digestion from reduced and alkylated proteins. They were subsequently purified, permethylated and finally measured by MALDI-TOF-MS. An exemplary annotated spectrum obtained from reference serum tube F is shown in S1 Fig.

Four of the detected N-glycans were high-mannose structures, seven fucosylated asialylated complex-type glycans, fourteen were complex-type glycans partially or fully capped with sialic acids and fourteen were fucosylated complex N-glycans with sialic acids (S2 Table).

**Fig 2:**
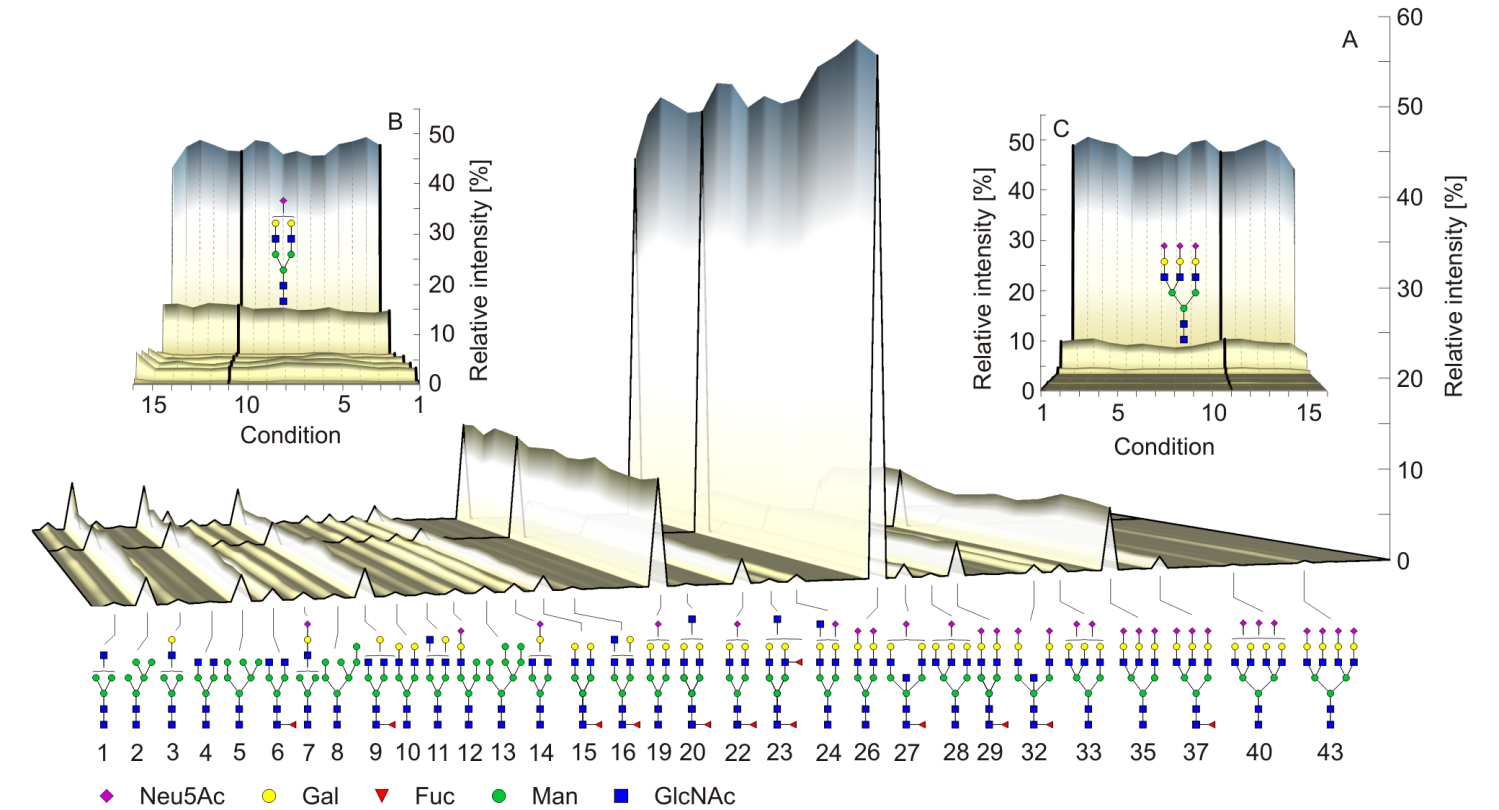
Results of MALDI-TOF-MS analysis. Average MALDI-TOF-MS N-glycan (A) relative intensities from 10 healthy donors under 16 preanalytical conditions (Fig 1). The serum and plasma standard conditions are marked with thick lines. Small increase of N-glycans having low masses (B) can be observed for hemolysed samples (condition 16), while N-glycans having high masses show a small decrease (C).

Relative intensities of 46 glycans (H = hexose, N = N-acetylhexosamine, S = Sialic acid, F = fucose), normalized to the sum of all glycans and means were calculated for triplicates (n = 465, Fig 2). The majority of the detected N-glycans was of complex-type (93.6% ± 3.7, 93.2% ± 2.8; mean ± SD for reference plasma and serum, respectively), followed by high-mannose (5.7% ± 3.5, 6.2% ± 2.6) and hybrid-type (0.7% ± 0.3%, 0.6 ± 0.2). Further differentiation of complex type N-glycans showed that diantennary N-glycans were the most abundant structures (72.5% ± 5.1, 72.8% ± 4.2; mean ± SD for reference plasma and serum, respectively), followed by triantennary (10.1% ± 3.6, 9.3% ± 4.0), monoantennary (6.9% ± 3.1, 6.9% ± 3.7) and tetraantennary (0.9% ± 0.3, 0.7% ± 0.4) N-glycans. Relative intensities of all structures for standard plasma and serum samples are shown in S3 Table.

**Fig 3:**
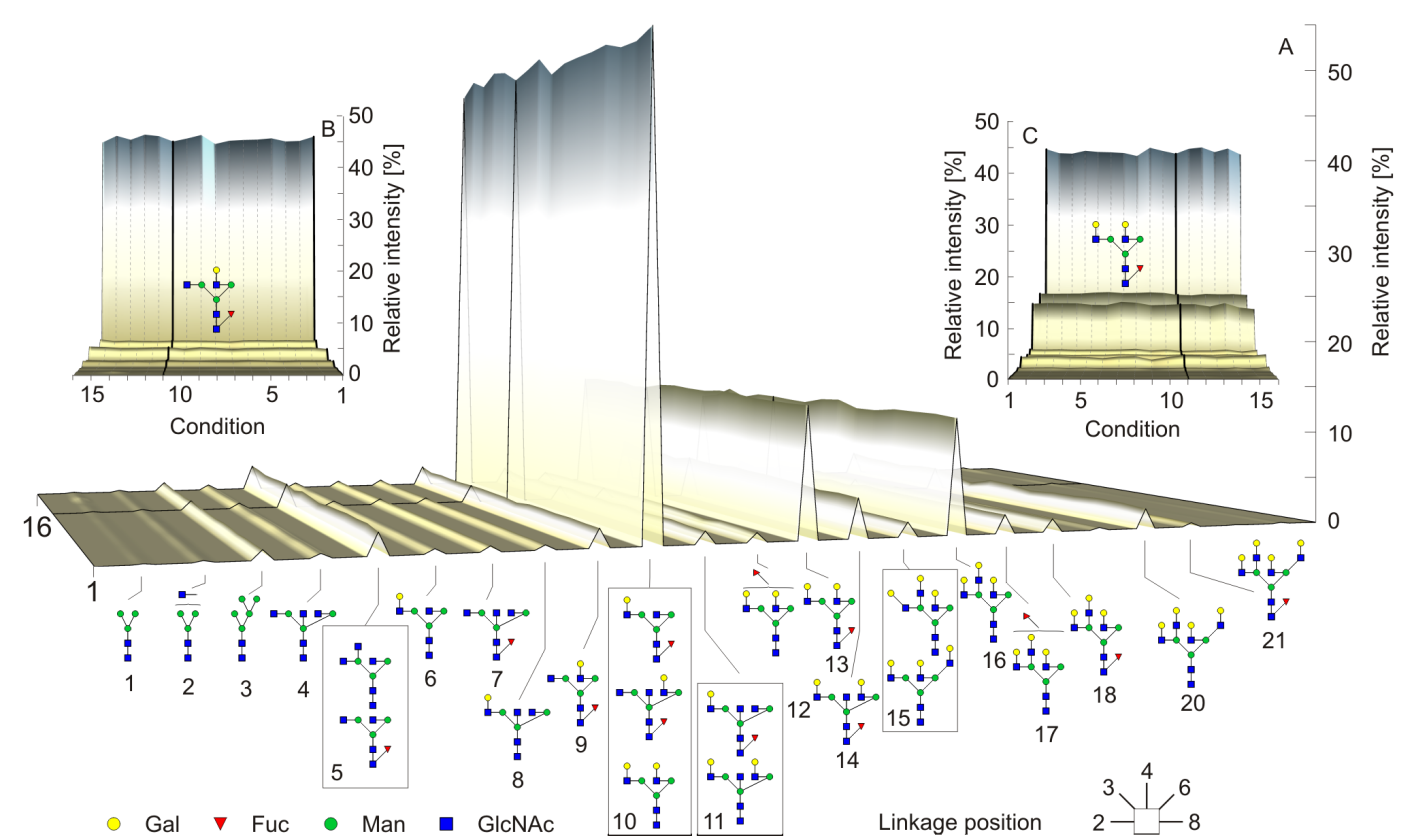
Results of CE-LIF analysis. Average CE-LIF N-glycan profiles (A) from 10 healthy donors under various preanalytical conditions. The conditions are numbered as mentioned in Fig 1. The serum and plasma standard conditions are marked with thick lines. Relative intensities of lower mass (B) and higher mass N-glycans (C) show the similar results throughout various conditions.

N-Glycans were released by PNGase F digestion from human serum or plasma, desialylated, labeled with APTS and measured by CE-LIF (Fig 3). This type of analysis enables the identification of N-glycan linkage and positional isomers, which is not possible by MALDI-TOF-MS analysis alone, however, the information about sialylation is lost as sialic acids are cleaved prior to analysis. Nevertheless, some glycan structures co-migrate by CE-LIF, leading to 23 glycan peaks. Their relative intensities were determined for each sample and means were calculated for duplicates (n = 310). The standard Oxford notation style was used for CE-LIF results due to its simplicity even for more complex structures. Explanation of naming and the corresponding CFG notations are depicted in S4 Table.

### N-Glycan profiling of preanalytical deviations by MALDI-TOF-MS and CE-LIF

For MALDI-TOF-MS we detected statistically significant differences for relative intensities of 19 glycans, four of those had a relative intensity > 1%. Among those 19 structures, four were high-mannose structures (N2H5, N2H6, N2H7, N2H8) and four asialylated complex-type structures (N3H4, N4H3, N4H5 and N5H4). Eight sialylated structures were significantly different. Five of them were monosialylated (N3H4S1, N3H5S1, N4H4S1, N4H5S1 and N5H6S1), two disialylated (N4H5S2, N5H6S2) and one tetrasialylated (N6H7S4). We detected no differences in relative intensities of trisialylated N-glycans. Two fucosylated structures (N5H4F1, N5H5F2) and only one from fourteen fucosylated sialylated structures (N6H7S4F1) were significantly different (S3A Table).

For the CE-LIF measurements 19 glycan peaks out of 23 were significantly different according to the Friedman’s test, from which ten had relative areas > 1% (S4A Table). It should be noted that the Friedman’s test detects differences between all the tested conditions, not only deviations from the standard procedures. Moreover, affected glycans generally had relative intensities under 1%.

From these results, we can conclude that high-mannose glycans appear to be the most sensitive N-glycans throughout all conditions, while fucosylated sialylated structures remain rather stable even during improper pre-analytical processing.

#### Effect of conditions before centrifugation

Three different conditions were used to test the effect of delayed centrifugation. Samples were kept at up-right position at room temperature for 2 hours and 6 hours and in the refrigerator (4–8°C) for 2 hours. We expected to observe changes caused by the activity of enzymes, such as sialidases. However, we did not detect any statistically significant change in relative intensities of the tested glycans caused by the delays in centrifugation when MALDI-TOF-MS and CE-LIF methods were used (S3B and S4B Tables).

#### Effect of long time storage and storage temperature

Two different storage temperatures were tested and compared to the standard preanalytical conditions. MALDI-TOF-MS measurements did not show statistically significant differences for samples stored for a period longer than 2 months neither at -20°C nor at -80°C. However, the CE-LIF measurements displayed significant differences between the standard serum preanalytical conditions and the non-standard conditions, namely cFA2BG2 (4.54%, 4.06–5.62), Z = -2.803, p = .005 for two months long storage at -80°C, and aF3A4G4 (0.06%, 0.05–0.08), Z = -2.803, p = .005 for two months storage at -20°C.

#### Effect of collection tube additives

There were no significant differences between the standard serum preanalytical conditions and the non-standard conditions when MALDI-TOF-MS was used, while the CE-LIF glycan peak containing structures A3G3[3] + A3[6]G3 (1.58%, 1.45–1.73), Z = -2.803, p = .005 was significantly increased for serum gel tube.

MALDI-TOF-MS measurements of plasma samples detected two structures, which were significantly decreased in EDTA plasma [N3H5S1 (0.43%, 0.31–0.49), Z = -2.803, p = .005; N5H5F2 (0.28%, 0.14–0.43), Z = -2.803, p = .005] and heparin plasma samples [N3H5S1 (0.37%, 0.27–0.49), Z = -2.803, p = .005; N5H5F2 (0.22%, 0.18–0.40), Z = -2.599, p = .009] compared to the standard plasma condition.

Additionally, six glycan peaks were significantly different between the standard plasma preanalytical condition and heparin for CE-LIF measurements. Three peaks [M3 (0.39%, 0.21–0.52), Z = -2.599, p = .009; A3G3[3] + A3[6]G3 (1.53%, 1.40–1.70), Z = -2.803, p = .005; and aFA3G3 (1.82%, 0.77–2.94), Z = -2.701, p = .007] were increased, and three peaks [FA2 + A3 (2.54%, 2.04–2.82), Z = -2.701, p = .007; A2[3]G1 (0.50%, 0.42–0.54), Z = -2.803, p = .005; and FA2B (0.56%, 0.53–0.64), Z = -2.701, p = .007] were decreased.

#### Effect of hypotonic conditions on the N-glycome

Mild hemolysis was induced by addition of deionized water to the sample. We did not detect any statistically significant differences between hemolysed samples and the reference plasma by MALDI-TOF-MS measurement, however the sample hemolysed by hypotonic conditions differed from the standard plasma condition in relative areas of A2B3G1 (0.59%, 0.54–0.62), Z = -2.701, p = .007, which was significantly increased, and A3G3 (11.3%, 9.24–13.23), Z = -2.803, p = .005, which was significantly decreased (S4B Table) as determined by CE-LIF.

#### Effect of overnight shaking at RT on the N-glycome

Strong hemolysis was induced in plasma samples from five donors by extreme conditions, *e*.*g*., mixing at 1400 rpm at RT overnight. We performed Wilcoxon signed-rank tests with Bonferroni correction applied (p < .01) to compare these samples to standard samples from the same individuals. Although samples were hemolysed, no statistically significant differences between the relative intensities of glycan structures were observed neither for MALDI-TOF-MS nor for CE-LIF measurements (see S3C and S4C Tables).

#### Correlation between hemolysis and N-glycome

Correlation analysis was performed to assess the relationship between relative intensities of individual glycans as well as calculated traits and the degree of hemolysis, which is presented as the concentration of free hemoglobin in mg/dl. The correlations are described using empirical classifications of interpreting correlation strength by using r proposed by Evans [39]. Two different types of hemolysis (tube K) were tested: hemolysis by hypotonic conditions (condition 15, n = 10) and hemolysis caused by combination of factors, *e*.*g*. mixing at 1400 rpm at RT overnight (condition 16, n = 5).

**Fig 4:**
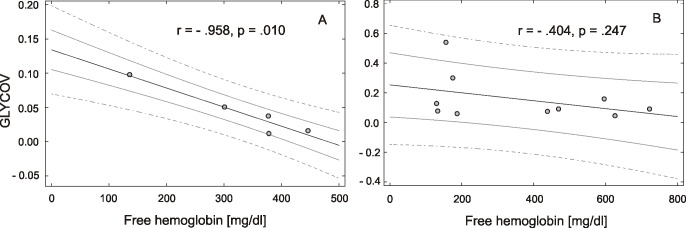
Scatterplot showing the correlation between free hemoglobin concentration and GLYCOV biomarker values. (A) samples hemolysed by mixing samples, (B) hemolysed by hypotonic conditions. Gray solid and dashed lines indicate the .75 and .95 confidence intervals, respectively. The GLYCOV score is a glycan score developed to diagnose ovarian cancer as follows: ratio (sum of relative intensities of (N5H6S3, N5H6S3F2, N6H7S3F1, N6H7S3F2, N6H7S4F1, N6H7S4F2, N6H7S4F3))/7*4/(sum of relative intensities of (N2H5, N2H6, N2H7 and N2H8).

We observed a statistically significant very strong negative correlation between relative intensity of N5H6F1 (r = -.917, n = 5, p = .028) and the degree of hemolysis in samples hemolysed by combination of factors. Even though other structures did not show a significant correlation between relative intensities and concentration of free hemoglobin, there was a highly significant very strong negative correlation with the value of glycan-based ovarian cancer biomarker (GLYCOV) (r = -.958, n = 5, p = .010), which is a score developed in our research group calculated as the ratio: (sum of relative intensities of (N5H6S3, N5H6S3F2, N6H7S3F1, N6H7S3F2, N6H7S4F1, N6H7S4F2, N6H7S4F3))/7*4/(sum of relative intensities of (N2H5, N2H6, N2H7 and N2H8)) (Fig 4A). It should be noted that none of the glycan structures that are used to calculate the biomarker GLYCOV showed statistically significant differences between the standard collection method and hemolysed sample when calculated individually. Nevertheless, the values of GLYCOV decreased upon hemolysis, which would presumably lead also to false negative measurements in ovarian cancer patients, indicating that hemolysed samples should not be considered used for biomarker assessments.

#### Correlation between hemolysis by hypotonic conditions and N-glycome

In hypotonically-hemolysed samples we found a statistically significant positive correlation between the concentration of free hemoglobin and relative intensities of two oligomannose structures and four asialo complex N-glycans of which two of these are fucosylated. There was a very strong positive correlation for N2H5 (r = .852, n = 10, p = .002), and a strong positive correlation for N2H6 (r = .715, n = 10, p = .020), N3H3 (r = .751, n = 10, p = .012), N3H4 (r = .770, n = 10, p = .009), N4H3F1 (r = .727, n = 10, p = .012) and N4H4F1 (r = .634, n = 10, p = .049)], which is in agreement with results observed for GLYCOV. There were also strong negative correlations for two N-glycans [N4H5S1 (r = -.695, n = 10, p = .026); N5H6S2 (r = -.667, n = 10, p = .035)] (S2 Fig). However, there was no statistically significant correlation between the GLYCOV value and the degree of hemolysis (r = -.404, n = 5, p = .247) (Fig 4B), even though two structures, which were used for its calculation, *e*.*g*., N2H5 and N2H6 showed strong individual correlation.

There was no statistically significant correlation between relative areas of glycan peaks and the degree of hemolysis when measured by CE-LIF for neither type of hemolysed samples. It should be noted that when the glycome is measured by CE-LIF, most oligomannose structures overlap and therefore cannot be measured distinctly.

## Discussion

This study, which investigated for the first time 16 different preanalytical conditions prior to N-glycan analysis carried out by MALDI-TOF-MS (n = 465 samples) and CE-LIF (n = 310 samples), highlights slight differences that could influence results of glycan biomarker studies. While intra-individual and longitudinal stability of the glycome and methodological robustness were previously analyzed [22–24], the present study is the unconditional prerequisite before translating glycan biomarker discovery into clinical chemistry laboratories.

Using MALDI-TOF-MS, it was shown that modulation of oligomannose structures can occur during pre-analytical processing, which affects glycan biomarkers such as the GLYCOV score developed in our laboratory [11] as shown in this report. The increase of oligomannose structures could result from nascent intracellular glycoproteins released during cell lysis [40]. The modulations of complex-type and sialylated glycans could be a result of circulating exoglycosidase activity [41, 42]. In this study, *in vitro* hemolysis was successfully induced by using extreme conditions after blood withdrawal, while no significant *in vivo* hemolysis was observed, since samples were taken from young healthy individuals. It was previously shown that *in vitro* hemolysis, one of the leading causes of preanalytical errors, is more likely to occur when sampling is performed in tubes filled below halfway in individuals older than 63 years [43, 44].

Using CE-LIF, it was shown that long time storage of deep frozen samples at -20°C or -80°C exerted only a minor influence on the glycome. Results were slightly different from MALDI-TOF-MS for two reasons. First, sialic acids were not investigated here and second, most oligomannose N-glycans co-migrate with other human blood N-glycans, preventing their analysis.

This study demonstrated that the glycome is more robust than other analytes such as proteins and peptides, for which preanalytical errors lead to non-reproducibility of ground-breaking publications [45] and to commercial failures in ovarian cancer diagnosis, such as lysophosphatidic acid as a marker for ovarian cancer [46]. Importantly, Diamandis and coworkers later discovered that lysophosphatidic acid was leaking from blood cells, thereby introducing preanalytical biases [47]. Nonetheless, it should be taken into account that our data were derived from healthy individuals. Thus, preanalytical analyses, as performed in the present study, are additionally warranted under specified disease conditions.

In this work, it could be shown that the glycome is rather stable in serum and plasma as short time delay between centrifugation at various temperatures seemed to have a minor effect on results. In addition, the glycome was stable upon storage at 4°C for at least 48 hours after centrifugation and aliquoting, which is an important information considering future diagnostic applications in clinical laboratories. However, it should be emphasized that hemolysis, a frequently seen clinical phenomenon in routine blood withdrawal procedures, affects relative intensities of certain glycans, which could lead to false negative (or positive) results in glycan biomarker studies and facilitate wrong clinical interpretations.

## Supporting information

S1 Fig MALDI-TOF-MS N-glycome profile obtained after PNGase F enzymatic release from plasmatic proteins, purification and permethylation. Measurements were performed in positive-ion mode. All ions are present in their sodiated form [M+Na]^+^. Structures are depicted following the CFG notation.
(PDF)

S2 Fig Scatter plots of free hemoglobin concentration and rel. intensities of N-glycans. (PDF)

S1 File Complete data of MALDI-TOF-MS and CE-LIF measurements. (XLSX)

S1 Table Masses of building blocks used for targeted data extraction by a Python script. (PDF)

S2 Table N-Glycans sorted by glycan type. (PDF)

S3 Table Results of MALDI-TOF-MS measurements and statistical analysis. Relative intensities of individual N-glycans (median, IQR) were tested for differences between all groups (n = 15) (a) by Friedman’s test and p-values < .05 were considered statistically significant (marked in red field). Only the structures, which were detected as significantly different by Friedman’s test, were further analysed by a Wilcoxon's pot-hoc test to identify whether such differences occurred as a result of deviation from the standard procedure. (b) Wilcoxon's test was used to compare standard conditions to all the altered conditions for plasma and serum separately. Statistical tests between two non-standard conditions were not performed, since these were not relevant for this study. Nine tests were performed for serum conditions, resulting in a Bonferroni corrected p value of .0056. For plasma conditions five tests were performed, including (c) Wilcoxon's test where results from only 5 individuals were compared, therefore the Bonferroni corrected p value for plasma samples was .01.
(PDF)

S4 Table Results of CE-LIF measurements and statistical analysis. Relative intensities of individual N-glycans (median, IQR) were tested for differences between all groups (n = 15) (a) by Friedman’s test and p-values < .05 were considered statistically significant (marked red). Only the structures, which were detected as significantly different by Friedman’s test, were further analysed by a Wilcoxon's post-hoc test to identify if such differences occurred as a result of deviation from the standard procedure. (b) Wilcoxon's test was used to compare standard conditions with the altered conditions for plasma and serum separately. Statistical tests between two non-standard conditions were not performed, since these are not relevant for this study. Nine tests were performed for serum conditions, resulting in a Bonferroni corrected p value of .0056. For plasma conditions five tests were performed including (c) Wilcoxon's test where results from only 5 individuals were compared, therefore the Bonferroni corrected p value for plasma samples was .01. The Oxford notations are: pentasaccharide core (A0) consists of three mannose residues and two N-acetylglucosamines (GlcNAc); F core fucose; aF antennary fucose; Ax, number GlcNAc attached to the core; B, bisecting GlcNAc; Gx, number of β1–4 linked galactose (G) on antennae; [3]G1 and [6]G1 indicates that the galactose is on the antenna of the α1–3 or α1–6 mannose. The [x] indicate the linkage type: in A2[3]G1 the galactose is linked to the α1–3 mannose, and in A3[2,2,6] the N-acetylglucosamines are β1-2-, β1-2-, and β1-6-linked to the trimannosyl core. In A3G3[3], the [3] indicates a β1–3 linkage between terminal G and GlcNAc.
(PDF)
