# Supplementary material for: The effect of blood sampling and preanalytical processing on human N-glycome
Source: PLoS One. 2018 Jul 11;13(7):e0200507. doi: 10.1371/journal.pone.0200507 (PMC6040761; doi:10.1371/journal.pone.0200507)
Supplement: S1 Fig — Measurements were performed in positive-ion mode. All ions are present in their sodiated form [M+Na]+. Structures are depicted following the CFG notation. (PDF) [file pone.0200507.s001.pdf]

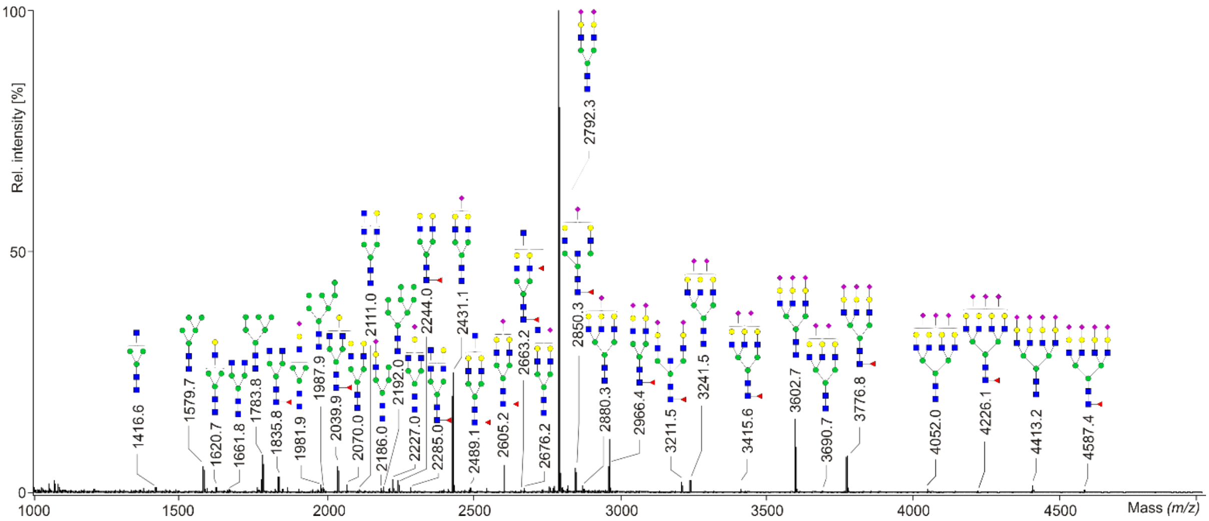


**S1 Fig.** MALDI-TOF-MS N-glycome profile obtained after PNGase F enzymatic release from plasmatic proteins, purification and permethylation. Measurements were performed in positive-ion mode. All ions are present in their sodiated form $[M+Na]^+$. Structures are depicted following the CFG notation.
