# Supplementary figures and images for: The effect of blood sampling and preanalytical processing on human N-glycome
Source: PLoS One. 2018 Jul 11;13(7):e0200507. doi: 10.1371/journal.pone.0200507 (PMC6040761; doi:10.1371/journal.pone.0200507)

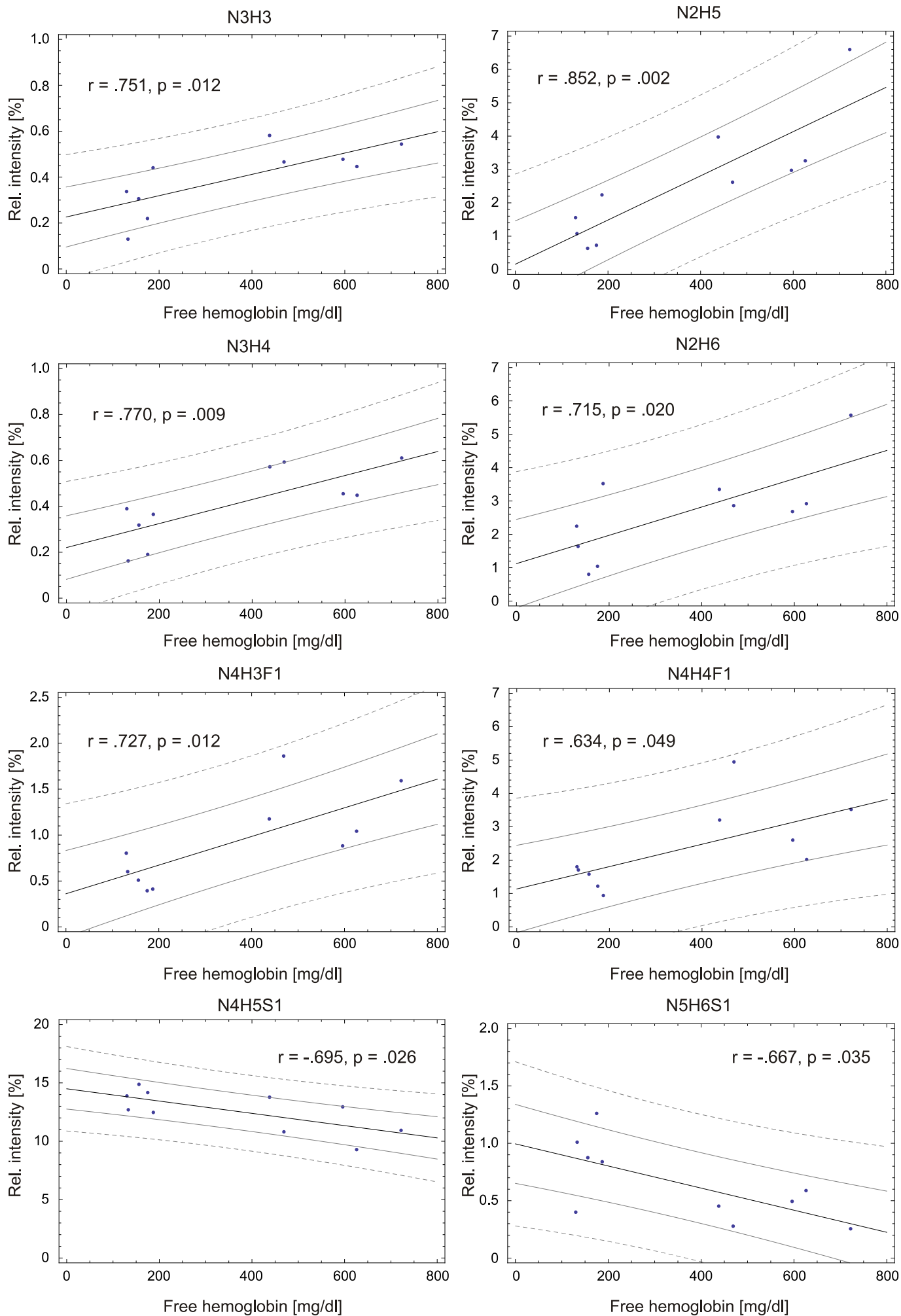

**S2 Fig.** Scatter plots of free hemoglobin concentration and rel.intensities of N-glycans

Supplement: S2 Fig — (PDF) [file pone.0200507.s002.pdf]
