# Supplementary material for: The effect of blood sampling and preanalytical processing on human N-glycome
Source: PLoS One. 2018 Jul 11;13(7):e0200507. doi: 10.1371/journal.pone.0200507 (PMC6040761; doi:10.1371/journal.pone.0200507)
Supplement: S1 Table — (PDF) [file pone.0200507.s004.pdf]

S1 Table. Masses of building blocks used for targeted data extraction by a Python script

	Mass [M + Na^+]	Carbons	Hydrogens	Nitrogens	Oxygens	Sulfurs
**Fucose**	174.089	8	14	0	4	0
**Hexose**	204.100	9	16	0	5	0
**N-acetylhexose**	245.126	11	19	1	5	0
**Sialic acid**	361.174	16	27	1	8	0
**Free end**	46.0419	2	8	0	1	0
