# Supplementary material for: The effect of blood sampling and preanalytical processing on human N-glycome
Source: PLoS One. 2018 Jul 11;13(7):e0200507. doi: 10.1371/journal.pone.0200507 (PMC6040761; doi:10.1371/journal.pone.0200507)
Supplement: S2 Table — (PDF) [file pone.0200507.s005.pdf]

S2 Table. N-Glycans sorted by glycan type

Glycan type	Structures
**Oligomannose**	N2H5, N2H6, N2H7, N2H8
**Fucosylated asialo**	N4H3F1, N4H4F1, N4H5F1, N5H4F1, N5H5F1, N5H5F2, N5H6F1
**Complex capped with sialic acids**	N3H4S1, N3H5S1, N4H4S1, N3H6S1, N4H5S1, N5H5S1, N4H5S2, N5H6S1, N5H5S2, N5H6S2, N5H6S3, N6H7S2, N6H7S3, N6H7S4
**Fucosylated complex capped with sialic acids**	N4H5S1F1, N5H5S1F1, N4H5S2F1, N5H6S1F1, N5H5S2F1, N5H6S2F1,N5H6S3F1, N6H7S2F1, N5H6S3F2, N6H7S3F1, N6H7S3F2, N6H7S4F1,N6H7S4F2, N6H7S4F3
**Afucosylated neutral complex**	N3H3, N3H4, N4H3, N4H5, N5H4, N5H5, N5H6
