# Supplementary material for: The effect of blood sampling and preanalytical processing on human N-glycome
Source: PLoS One. 2018 Jul 11;13(7):e0200507. doi: 10.1371/journal.pone.0200507 (PMC6040761; doi:10.1371/journal.pone.0200507)
Supplement: S3 Table — Relative intensities of individual N-glycans (median, IQR) were tested for differences between all groups (n = 15) (a) by Friedman’s test and p-values < .05 were considered statistically significant (marked in red field). Only the structures, which were detected as significantly different by Friedman’s test, were further analysed by a Wilcoxon's pot-hoc test to identify whether such differences occurred as a result of deviation from the standard procedure. (b) Wilcoxon's test was used to compare standard conditions to all the altered conditions for plasma and serum separately. Statistical tests between two non-standard conditions were not performed, since these were not relevant for this study. Nine tests were performed for serum conditions, resulting in a Bonferroni corrected p value of .0056. For plasma conditions five tests were performed, including (c) Wilcoxon's test where results from only 5 individuals were compared, therefore the Bonferroni corrected p value for plasma samples was .01. (PDF) [file pone.0200507.s006.pdf]

S3 Table. Results of MALDI-TOF-MS measurements and statistical analysis. Relative intensities of individual N-glycans (median, IQR) were tested for differences between all groups (n=15) (a) by Friedman's test and p < .05 were considered statistically significant. Only the structures, which were detected as significantly different by Friedman's test were further analysed by a Wilcoxon's pot-hoc test to identify whether such differences occurred as a result of deviation from the standard procedure. (b) Wilcoxon's test was used to compare standard conditions to all the altered conditions for plasma and serum separately. Statistical tests between two non-standard conditions were not performed, since these are not relevant for this study. Nine tests were performed for serum conditions, resulting in a Bonferroni corrected p value of .0056. For plasma conditions five tests were performed, including (c) Wilcoxon's test where results from only 5 individuals were compared, therefore the Bonferroni corrected p value for plasma samples was .01.

Structure				Friedman's test[a]		Wilcoxon's test[b]															Wilcoxon's test[c]		
Peak	Desialylated CFG notation	CFG notation			All conditions		1 Serum ref.	2 6h RT	3 2h RT	4 2h 4°C	5 6h after	6 gel 6h after	7 2 months -80°C	8 2 months -20°C	9 24h 4°C	11 Plasma ref. n=10	12 Vacuum	13 EDTA	14 Heparin	15 Hemolysis water		11 Plasma ref. n=5	16 Hemolysis combined
1	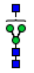	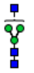	N3H3	Median	0.35	Median	0.33	0.30	0.26	0.35	0.27	0.24	0.54	0.44	0.37	0.45	0.36	0.29	0.40	0.44	Median	0.60	0.63
			m/z	IQR	0.23	IQR	0.19	0.12	0.13	0.28	0.24	0.20	0.22	0.23	0.25	0.22	0.23	0.23	0.19	0.28	IQR	0.45	0.50
			1416.6		0.56		0.47	0.49	0.46	0.44	0.40	0.33	0.77	0.69	0.55	0.60	0.66	0.47	0.63	0.49		0.67	1.33
				$\chi^2(14)$	20.610	Z	-	-	-	-	-	-	-	-	-	-	-	-	-	-	Z	-	-0.944
				p value	0.112	p value	-	-	-	-	-	-	-	-	-	-	-	-	-	-	p value	-	0.345
2	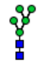	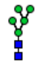	N2H5	Median	2.43	Median	2.40	2.35	1.80	2.40	2.48	2.36	2.94	3.94	3.70	2.17	2.02	2.31	2.23	2.43	Median	3.16	4.88
			m/z	IQR	1.31	IQR	1.18	0.97	0.87	1.59	1.68	1.20	1.86	1.53	1.26	1.02	1.30	1.12	1.36	0.99	IQR	2.17	4.17
			1579.7		4.25		3.99	4.20	4.01	4.19	4.84	3.93	6.37	5.26	5.56	3.51	3.89	3.86	3.59	3.44		5.48	7.95
				$\chi^2(14)$	31.530	Z	-	-0.255	-0.968	-1.070	-1.274	-0.561	-1.988	-2.191	-1.784	-	-0.663	-0.866	-0.764	-0.459	Z	-	-2.023
				p value	**0.005**	p value	-	0.799	0.333	0.285	0.203	0.575	0.047	0.028	0.074	-	0.508	0.386	0.445	0.646	p value	-	0.043
3	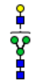	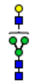	N3H4	Median	0.40	Median	0.43	0.30	0.26	0.41	0.30	0.23	0.58	0.60	0.38	0.47	0.41	0.25	0.43	0.42	Median	0.58	0.70
			m/z	IQR	0.22	IQR	0.24	0.13	0.12	0.27	0.21	0.18	0.37	0.25	0.22	0.18	0.21	0.22	0.22	0.29	IQR	0.50	0.45
			1620.7		0.54		0.55	0.54	0.44	0.55	0.42	0.45	0.74	0.72	0.61	0.59	0.66	0.40	0.52	0.58		0.74	1.39
				$\chi^2(14)$	36.760	Z	-	-0.968	-2.497	-0.153	-1.682	-2.599	-1.580	-0.968	-0.357	-	-1.478	-0.663	-1.580	-0.459	Z	-	-0.135
				p value	**0.001**	p value	-	0.333	0.013	0.878	0.093	0.009	0.114	0.333	0.721	-	0.139	0.508	0.114	0.646	p value	-	0.893
4	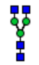	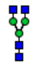	N4H3	Median	0.11	Median	0.10	0.09	0.08	0.14	0.10	0.09	0.16	0.16	0.10	0.12	0.14	0.09	0.14	0.13	Median	0.15	0.18
			m/z	IQR	0.08	IQR	0.08	0.02	0.04	0.07	0.08	0.07	0.09	0.08	0.07	0.07	0.08	0.09	0.06	0.08	IQR	0.12	0.11
			1661.8		0.17		0.15	0.19	0.17	0.18	0.13	0.13	0.22	0.23	0.20	0.15	0.23	0.14	0.16	0.15		0.19	0.33
				$\chi^2(14)$	25.970	Z	-	-0.866	-0.663	-0.255	-0.866	-1.478	-2.090	-1.376	-0.051	-	-1.886	-0.357	-1.172	-0.357	Z	-	-1.214
				p value	**0.026**	p value	-	0.386	0.508	0.799	0.386	0.139	0.037	0.169	0.959	-	0.059	0.721	0.241	0.721	p value	-	0.225
5	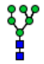	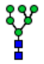	N2H6	Median	2.73	Median	2.64	2.44	1.99	2.67	3.07	2.42	3.42	3.52	3.80	2.07	2.26	2.40	2.86	2.77	Median	2.46	5.07
			m/z	IQR	1.65	IQR	1.72	1.41	1.30	1.80	1.99	1.70	2.28	1.75	1.50	1.35	1.76	1.50	2.04	1.49	IQR	2.07	3.31
			1783.8		3.85		3.56	3.64	3.68	3.76	4.51	3.96	5.56	4.88	5.33	2.93	3.66	3.16	3.96	3.39		4.61	7.15
				$\chi^2(14)$	28.770	Z	-	-0.255	-1.070	-1.172	-1.478	-0.561	-2.090	-2.090	-1.478	-	-0.764	-2.191	-0.866	-1.172	Z	-	-1.753
				p value	**0.011**	p value	-	0.799	0.285	0.241	0.139	0.575	0.037	0.037	0.139	-	0.445	0.028	0.386	0.241	p value	-	0.080
6	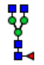	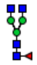	N4H3F1	Median	0.96	Median	0.80	0.96	0.76	1.01	0.95	0.92	0.91	0.97	1.34	1.05	0.93	1.00	0.79	0.84	Median	1.38	1.41
			m/z	IQR	0.52	IQR	0.49	0.24	0.45	0.51	0.57	0.33	0.61	0.61	0.40	0.54	0.61	0.51	0.46	0.48	IQR	1.12	1.21
			1835.8		1.50		1.68	1.61	1.50	1.51	1.33	1.41	1.60	1.43	2.02	1.47	1.58	1.35	1.54	1.28		2.18	2.87
				$\chi^2(14)$	12.720	Z	-	-	-	-	-	-	-	-	-	-	-	-	-	-	Z	-	-0.674
				p value	0.549	p value	-	-	-	-	-	-	-	-	-	-	-	-	-	-	p value	-	0.500
7	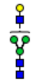	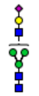	N3H4S1	Median	0.56	Median	0.62	0.46	0.41	0.62	0.40	0.44	0.80	0.75	0.74	0.57	0.52	0.56	0.54	0.72	Median	0.76	0.60
			m/z	IQR	0.42	IQR	0.48	0.35	0.25	0.41	0.30	0.31	0.70	0.48	0.41	0.36	0.46	0.43	0.42	0.56	IQR	0.55	0.54
			1981.9		0.78		0.84	0.51	0.58	1.01	0.79	0.57	0.94	0.82	0.99	0.78	0.86	0.67	0.69	0.86		0.86	1.00
				$\chi^2(14)$	31.160	Z	-	-1.886	-2.395	-0.051	-1.070	-2.599	-1.580	-0.357	-0.153	-	-1.172	-0.968	-0.255	-0.663	Z	-	-0.405
				p value	**0.005**	p value	-	0.059	0.017	0.959	0.285	0.009	0.114	0.721	0.878	-	0.241	0.333	0.799	0.508	p value	-	0.686

Structure				Friedman's test[a]		Wilcoxon's test[b]															Wilcoxon's test[c]		
Peak	Desialylated CFG notation	CFG notation			All conditions		1 Serum ref.	2 6h RT	3 2h RT	4 2h 4°C	5 6h after	6 gel 6h after	7 2 months -80°C	8 2 months -20°C	9 24h 4°C	11 Plasma ref. n=10	12 Vacuum	13 EDTA	14 Heparin	15 Hemolysis water		11 Plasma ref. n=5	16 Hemolysis combined
8	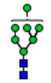	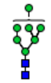		Median	0.45	Median	0.39	0.42	0.41	0.50	0.56	0.40	0.64	0.64	0.59	0.32	0.40	0.39	0.49	0.37	Median	0.35	0.66
			**N2H7**	IQR	0.34	IQR	0.31	0.29	0.23	0.38	0.38	0.33	0.46	0.48	0.35	0.24	0.30	0.28	0.37	0.29	IQR	0.23	0.47
			m/z		0.64		0.53	0.55	0.57	0.61	0.71	0.54	0.80	0.72	0.87	0.45	0.54	0.49	0.55	0.52		0.69	0.89
			1987.9	$\chi^2(14)$	36.470	Z	-	-0.051	-0.255	-1.172	-1.580	-0.357	-2.395	-2.395	-1.376	-	-1.886	-2.191	-1.172	-1.886	Z	-	-1.753
				p value	**0.001**	p value	-	0.959	0.799	0.241	0.114	0.721	0.017	0.017	0.169	-	0.059	0.028	0.241	0.059	p value	-	0.080
9	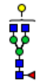	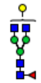		Median	2.32	Median	1.89	2.21	1.93	2.33	2.34	2.21	2.51	2.52	2.83	2.46	2.13	2.29	1.84	1.91	Median	3.16	3.01
			**N4H4F1**	IQR	1.58	IQR	1.54	0.68	1.09	1.50	1.48	1.04	1.83	1.93	0.83	1.58	1.53	1.51	1.25	1.49	IQR	2.66	2.69
			m/z		3.52		3.45	3.98	3.69	3.29	3.02	3.65	3.21	2.89	4.75	3.65	4.01	2.95	3.57	3.28		5.59	7.19
			2039.9	$\chi^2(14)$	16.650	Z	-	-	-	-	-	-	-	-	-	-	-	-	-	-	Z	-	-0.135
				p value	0.275	p value	-	-	-	-	-	-	-	-	-	-	-	-	-	-	p value	-	0.893
10	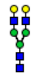	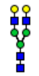		Median	0.36	Median	0.32	0.30	0.30	0.38	0.33	0.28	0.50	0.45	0.45	0.36	0.34	0.34	0.31	0.33	Median	0.44	0.38
			**N4H5**	IQR	0.28	IQR	0.25	0.19	0.26	0.29	0.27	0.25	0.44	0.33	0.36	0.28	0.32	0.27	0.23	0.29	IQR	0.27	0.30
			m/z		0.47		0.52	0.48	0.42	0.48	0.48	0.45	0.58	0.51	0.53	0.45	0.39	0.44	0.43	0.44		0.54	0.73
			2070.0	$\chi^2(14)$	24.310	Z	-	-0.968	-1.070	-0.561	-0.357	-1.580	-2.090	-1.376	-0.866	-	-0.459	-1.376	-0.561	-0.561	Z	-	-0.944
				p value	**0.042**	p value	-	0.333	0.285	0.575	0.721	0.114	0.037	0.169	0.386	-	0.646	0.169	0.575	0.575	p value	-	0.345
11	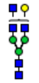	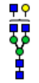		Median	0.19	Median	0.18	0.15	0.16	0.19	0.22	0.17	0.26	0.23	0.25	0.16	0.16	0.19	0.17	0.18	Median	0.18	0.20
			**N5H4**	IQR	0.14	IQR	0.16	0.13	0.13	0.14	0.13	0.14	0.19	0.17	0.17	0.12	0.13	0.13	0.16	0.13	IQR	0.16	0.17
			m/z		0.26		0.25	0.27	0.23	0.26	0.32	0.26	0.40	0.31	0.37	0.25	0.23	0.23	0.27	0.23		0.37	0.46
			2111.0	$\chi^2(14)$	31.090	Z		-1.172	-1.478	-0.663	-0.357	-1.274	-1.274	-0.357	-0.663	-	-0.764	-0.459	-0.051	-0.153	Z	-	-1.214
				p value	**0.005**	p value		0.241	0.139	0.508	0.721	0.203	0.203	0.721	0.508	-	0.445	0.646	0.959	0.878	p value	-	0.225
12	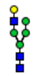	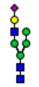		Median	0.45	Median	0.45	0.38	0.35	0.44	0.43	0.41	0.37	0.38	0.53	0.51	0.50	0.43	0.37	0.71	Median	0.88	0.83
			**N3H5S1**	IQR	0.34	IQR	0.39	0.30	0.30	0.36	0.34	0.29	0.34	0.33	0.38	0.44	0.33	0.31	0.27	0.55	IQR	0.51	0.70
			m/z		0.58		0.59	0.59	0.59	0.61	0.51	0.50	0.44	0.48	0.61	0.90	0.85	0.49	0.49	0.89		0.96	1.14
			2186.0	$\chi^2(14)$	45.260	Z	-	-1.172	-0.968	-0.153	-1.478	-1.376	-1.886	-1.478	-0.357	-	-0.051	-2.803	-2.803	-1.376	Z	-	-0.944
				p value	**< 0.0001**	p value	-	0.241	0.333	0.878	0.139	0.169	0.059	0.139	0.721	-	0.959	**0.005**	**0.005**	0.169	p value	-	0.345
13	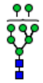	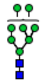		Median	0.71	Median	0.63	0.66	0.69	0.72	0.89	0.73	1.00	0.87	0.93	0.46	0.66	0.59	0.69	0.66	Median	0.46	0.99
			**N2H8**	IQR	0.53	IQR	0.48	0.44	0.36	0.57	0.56	0.50	0.79	0.72	0.58	0.38	0.47	0.51	0.58	0.42	IQR	0.38	0.73
			m/z		0.99		0.81	0.77	0.90	1.02	1.09	0.87	1.10	1.14	1.29	0.61	0.80	0.78	0.89	0.82		0.94	1.31
			2192.0	$\chi^2(14)$	36.580	Z	-	-0.153	-0.051	-0.866	-1.376	-0.663	-2.191	-2.497	-1.580	-	-1.784	-2.090	-1.580	-1.682	Z	-	-2.023
				p value	**0.001**	p value	-	0.878	0.959	0.386	0.169	0.508	0.028	0.013	0.114	-	0.074	0.037	0.114	0.093	p value	-	0.043
14	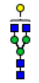	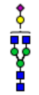		Median	0.71	Median	0.84	0.53	0.52	0.81	0.58	0.59	0.89	0.68	0.79	0.79	0.69	0.74	0.58	0.81	Median	0.76	0.64
			**N4H4S1**	IQR	0.54	IQR	0.57	0.41	0.48	0.37	0.33	0.36	0.71	0.58	0.58	0.63	0.59	0.54	0.48	0.67	IQR	0.60	0.49
			m/z		0.89		0.93	0.75	0.69	1.07	0.91	0.79	1.06	0.86	1.01	0.90	0.97	0.91	0.72	1.25		0.96	0.94
			2227.0	$\chi^2(14)$	36.140	Z	-	-2.191	-2.191	-0.153	-1.070	-2.599	-0.968	-0.866	-0.255	-	-0.459	-2.191	-0.866	-1.172	Z	-	-0.944
				p value	**0.001**	p value	-	0.028	0.028	0.878	0.285	0.009	0.333	0.386	0.799	-	0.646	0.028	0.386	0.241	p value	-	0.345
15	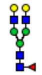	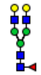		Median	1.27	Median	1.21	1.09	1.32	1.18	1.33	1.12	1.27	1.41	1.45	1.39	1.20	1.30	1.01	1.18	Median	1.67	1.55
			**N4H5F1**	IQR	0.99	IQR	0.95	0.69	0.91	0.89	1.06	0.96	1.21	1.26	0.84	1.08	1.07	1.02	0.74	0.97	IQR	1.39	1.33
			m/z		1.80		1.71	1.98	1.84	1.95	1.49	1.78	1.87	1.57	2.35	1.85	1.92	1.69	1.56	1.62		2.43	2.81
			2244.0	$\chi^2(14)$	14.470	Z	-	-	-	-	-	-	-	-	-	-	-	-	-	-	Z	-	-0.405
				p value	0.415	p value	-	-	-	-	-	-	-	-	-	-	-	-	-	-	p value	-	0.686
16	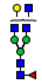	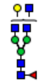		Median	0.25	Median	10.78	10.06	10.09	10.55	10.97	11.22	11.88	11.35	11.52	11.58	11.78	12.16	11.13	12.82	Median	0.25	0.25
			**N5H4F1**	IQR	0.20	IQR	9.55	9.62	9.23	9.14	9.41	9.50	9.81	9.46	9.79	10.47	10.71	10.78	9.90	10.90	IQR	0.18	0.22
			m/z		0.32		11.88	10.80	12.02	12.62	13.52	12.30	12.52	12.47	13.87	13.92	13.63	14.52	13.15	13.95		0.36	0.34
			2285.1	$\chi^2(14)$	33.570	Z	-	-1.478	-1.274	-1.376	-0.051	-1.682	-1.886	-1.376	-0.357	-	-0.051	-0.051	-0.866	-0.153	Z	-	-0.405
				p value	**0.002**	p value	-	0.139	0.203	0.169	0.959	0.093	0.059	0.169	0.721	-	0.959	0.959	0.386	0.878	p value	-	0.686

Structure				Friedman's test[a]		Wilcoxon's test[b]															Wilcoxon's test[c]		
Peak	Desialylated CFG notation	CFG notation			All conditions		1 Serum ref.	2 6h RT	3 2h RT	4 2h 4°C	5 6h after	6 gel 6h after	7 2 months -80°C	8 2 months -20°C	9 24h 4°C	11 Plasma ref. n=10	12 Vacuum	13 EDTA	14 Heparin	15 Hemolysis water		11 Plasma ref. n=5	16 Hemolysis combined
17	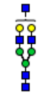	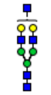		Median	0.06	Median	0.06	0.07	0.05	0.07	0.06	0.07	0.07	0.07	0.08	0.07	0.05	0.06	0.06	0.06	Median	0.06	0.09
			N5H5	IQR	0.05	IQR	0.04	0.05	0.04	0.05	0.05	0.05	0.05	0.06	0.05	0.04	0.05	0.04	0.04	0.04	IQR	0.04	0.05
			m/z		0.09		0.09	0.09	0.08	0.14	0.09	0.09	0.11	0.09	0.11	0.10	0.08	0.11	0.08	0.08		0.09	0.12
			2315.1	χ^2(14)	13.080	Z	-	-	-	-	-	-	-	-	-	-	-	-	-	-	Z	-	-1.753
				p value	0.520	p value	-	-	-	-	-	-	-	-	-	-	-	-	-	-	p value	-	0.080
18	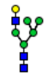	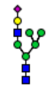		Median	0.07	Median	0.06	0.07	0.05	0.08	0.08	0.08	0.07	0.07	0.08	0.06	0.05	0.06	0.06	0.08	Median	0.05	0.08
			N3H6S1	IQR	0.05	IQR	0.05	0.04	0.04	0.05	0.06	0.05	0.06	0.05	0.05	0.04	0.04	0.04	0.05	0.06	IQR	0.04	0.06
			m/z		0.09		0.09	0.10	0.09	0.12	0.09	0.09	0.09	0.08	0.11	0.08	0.09	0.09	0.08	0.10		0.08	0.12
			2390.1	χ^2(14)	16.390	Z	-	-	-	-	-	-	-	-	-	-	-	-	-	-	Z	-	-1.753
				p value	0.290	p value	-	-	-	-	-	-	-	-	-	-	-	-	-	-	p value	-	0.080
19	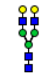	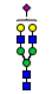		Median	11.11	Median	1.21	1.09	1.32	1.18	1.33	1.12	1.27	1.41	1.45	1.39	1.20	1.30	1.01	1.18	Median	11.76	11.31
			N4H5S1	IQR	9.91	IQR	0.95	0.69	0.91	0.89	1.06	0.96	1.21	1.26	0.84	1.08	1.07	1.02	0.74	0.97	IQR	10.38	11.09
			m/z		12.82		1.71	1.98	1.84	1.95	1.49	1.78	1.87	1.57	2.35	1.85	1.92	1.69	1.56	1.62		14.56	14.32
			2431.1	χ^2(14)	28.140	Z	-	-1.478	-0.663	-0.561	-1.070	-1.172	-0.866	-0.153	-1.376		-0.663	-0.866	-0.153	-0.051	Z	-	-0.135
				p value	**0.014**	p value	-	0.139	0.508	0.575	0.285	0.241	0.386	0.878	0.169		0.508	0.386	0.878	0.959	p value	-	0.893
20	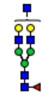	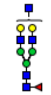		Median	0.18	Median	0.20	0.13	0.18	0.19	0.18	0.18	0.19	0.24	0.25	0.18	0.17	0.20	0.13	0.19	Median	0.12	0.16
			N5H5F1	IQR	0.12	IQR	0.10	0.09	0.11	0.09	0.13	0.12	0.11	0.11	0.16	0.11	0.14	0.11	0.10	0.11	IQR	0.08	0.10
			m/z		0.24		0.25	0.31	0.22	0.28	0.28	0.24	0.30	0.31	0.30	0.21	0.21	0.23	0.22	0.23		0.19	0.19
			2489.1	χ^2(14)	20.570	Z	-	-	-	-	-	-	-	-	-	-	-	-	-	-	Z	-	-0.674
				p value	0.113	p value	-	-	-	-	-	-	-	-	-	-	-	-	-	-	p value	-	0.500
21	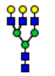	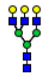		Median	0.01	Median	0.01	0.02	0.01	0.01	0.02	0.02	0.02	0.02	0.01	0.01	0.01	0.01	0.02	0.01	Median	0.01	0.02
			N5H6	IQR	0.01	IQR	0.00	0.01	0.01	0.00	0.01	0.01	0.00	0.01	0.01	0.01	0.00	0.01	0.00	0.00	IQR	0.01	0.00
			m/z		0.03		0.02	0.03	0.02	0.03	0.03	0.03	0.03	0.03	0.03	0.03	0.03	0.04	0.04	0.02		0.03	0.03
			2519.1	χ^2(14)	10.740	Z	-	-	-	-	-	-	-	-	-	-	-	-	-	-	Z	-	-0.135
				p value	0.706	p value	-	-	-	-	-	-	-	-	-	-	-	-	-	-	p value	-	0.893
22	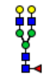	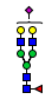		Median	2.52	Median	2.54	2.37	2.51	2.58	2.64	2.59	2.63	2.69	2.32	2.25	2.37	2.51	2.21	2.55	Median	2.14	1.90
			N4H5S1F1	IQR	1.98	IQR	1.88	1.75	2.13	1.98	2.08	1.99	1.96	1.86	2.10	2.07	1.95	1.97	1.77	1.94	IQR	1.80	1.68
			m/z		2.86		3.02	2.91	2.83	2.65	2.99	2.86	3.11	3.24	3.30	2.84	2.58	2.89	2.73	3.04		2.77	2.74
			2605.2	χ^2(14)	21.870	Z	-	-	-	-	-	-	-	-	-	-	-	-	-	-	Z		-1.214
				p value	0.081	p value	-	-	-	-	-	-	-	-	-	-	-	-	-	-	p value		0.225
23	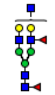	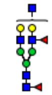		Median	0.30	Median	0.40	0.34	0.37	0.27	0.27	0.28	0.15	0.16	0.27	0.57	0.40	0.28	0.22	0.70	Median	0.55	0.41
			N5H5F2	IQR	0.19	IQR	0.26	0.23	0.20	0.14	0.20	0.20	0.14	0.12	0.20	0.44	0.22	0.14	0.18	0.56	IQR	0.41	0.41
			m/z		0.44		0.45	0.44	0.41	0.35	0.31	0.33	0.20	0.22	0.34	0.68	0.70	0.43	0.40	0.74		0.73	0.52
			2663.2	χ^2(14)	54.310	Z	-	-1.376	-0.459	-1.886	-2.191	-1.376	-2.497	-1.682	-1.682	-	-0.968	-2.803	-2.599	-1.274	Z	-	-1.214
				p value	**< 0.0001**	p value	-	0.169	0.646	0.059	0.028	0.169	0.013	0.093	0.093	-	0.333	**0.005**	**0.009**	0.203	p value	-	0.225
24	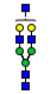	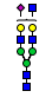		Median	0.58	Median	0.63	0.50	0.64	0.52	0.53	0.64	0.62	0.63	0.64	0.55	0.54	0.61	0.53	0.58	Median	0.51	0.43
			N5H5S1	IQR	0.43	IQR	0.41	0.46	0.40	0.29	0.43	0.40	0.45	0.43	0.50	0.39	0.44	0.43	0.41	0.46	IQR	0.39	0.36
			m/z		0.88		0.95	0.81	0.78	0.94	0.88	0.86	0.98	0.96	0.85	0.95	0.78	0.80	0.70	0.97		0.83	0.78
			2676.2	χ^2(14)	20.800	Z	-	-	-	-	-	-	-	-	-	-	-	-	-	-	Z	-	-0.405
				p value	0.107	p value	-	-	-	-	-	-	-	-	-	-	-	-	-	-	p value	-	0.686
25	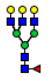	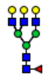		Median	0.02	Median	0.02	0.01	0.01	0.01	0.02	0.02	0.01	0.02	0.03	0.03	0.01	0.02	0.03	0.01	Median	0.02	0.03
			N5H6F1	IQR	0.01	IQR	0.01	0.01	0.01	0.01	0.01	0.01	0.00	0.01	0.01	0.01	0.00	0.01	0.01	0.01	IQR	0.01	0.01
			m/z		0.03		0.03	0.02	0.02	0.02	0.03	0.04	0.03	0.04	0.04	0.04	0.02	0.03	0.03	0.02		0.04	0.04
			2693.2	χ^2(14)	15.930	Z	-	-	-	-	-	-	-	-	-	-	-	-	-	-	Z	-	-0.405
				p value	0.318	p value	-	-	-	-	-	-	-	-	-	-	-	-	-	-	p value	-	0.686

Structure				Friedman's test[a]		Wilcoxon's test[b]															Wilcoxon's test[c]		
							1	2	3	4	5	6	7	8	9	11	12	13	14	15		11	16
Peak	Desialylated CFG notation	CFG notation			All conditions		Serum ref.	6h RT	2h RT	2h 4°C	6h after	gel 6h after	2 months -80°C	2 months -20°C	24h 4°C	Plasma ref. n=10	Vacuum	EDTA	Heparin	Hemolysis water		Plasma ref. n=5	Hemolysis combined
26	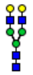	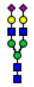		Median	53.90	Median	54.22	57.23	55.39	54.72	54.67	56.37	52.08	53.64	51.02	53.48	53.71	54.58	56.41	53.44	Median	49.46	47.29
			N4H5S2	IQR	50.80	IQR	52.00	51.21	51.42	52.16	51.43	51.59	48.87	50.27	47.76	48.94	49.38	49.52	53.41	51.00	IQR	45.90	41.70
			m/z		57.09		57.88	60.64	57.75	57.52	58.88	58.67	54.17	54.05	56.94	56.58	56.27	57.56	58.24	56.35		53.48	54.85
			2792.3	$\chi^2(14)$	29.530	Z	-	-1.274	-0.968	-0.153	-0.663	-1.682	-2.191	-1.274	-1.070	-	-0.968	-1.580	-1.274	-1.172	Z	-	-0.135
				p value	**0.009**	p value	-	0.203	0.333	0.878	0.508	0.093	0.028	0.203	0.285	-	0.333	0.114	0.203	0.241	p value	-	0.893
27	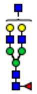	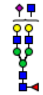		Median	1.18	Median	1.29	1.03	1.25	1.17	1.26	1.23	1.26	1.49	1.09	1.12	1.28	1.22	1.18	1.24	Median	1.10	1.17
			N5H5S1F1	IQR	0.84	IQR	0.75	0.70	0.81	0.70	1.05	0.74	0.96	0.89	0.84	0.81	0.92	0.96	0.83	0.76	IQR	0.77	0.43
			m/z		1.75		2.00	1.61	1.61	1.83	2.12	1.94	1.80	2.09	2.14	1.75	1.65	1.82	1.92	1.72		1.55	1.57
			2850.3	$\chi^2(14)$	15.090	Z	-	-	-	-	-	-	-	-	-	-	-	-	-	-	Z	-	-0.944
				p value	0.372	p value	-	-	-	-	-	-	-	-	-	-	-	-	-	-	p value	-	0.345
28	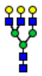	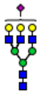		Median	0.32	Median	0.31	0.30	0.28	0.33	0.33	0.31	0.34	0.38	0.35	0.32	0.27	0.31	0.28	0.30	Median	0.34	0.20
			N5H6S1	IQR	0.26	IQR	0.28	0.24	0.23	0.26	0.29	0.23	0.29	0.35	0.28	0.23	0.24	0.25	0.19	0.22	IQR	0.28	0.17
			m/z		0.38		0.40	0.34	0.38	0.42	0.36	0.39	0.47	0.54	0.42	0.37	0.32	0.36	0.36	0.35		0.40	0.28
			2880.4	$\chi^2(14)$	31.520	Z	-	-1.172	-1.478	-0.255	-0.561	-1.376	-1.274	-2.090	-0.255	-	-0.866	-0.764	-0.561	-0.663	Z	-	-1.483
				p value	**0.005**	p value	-	0.241	0.139	0.799	0.575	0.169	0.203	0.037	0.799	-	0.386	0.445	0.575	0.508	p value	-	0.138
29	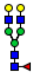	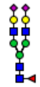		Median	3.24	Median	3.59	3.22	3.46	3.12	2.94	3.41	3.33	3.56	2.86	3.13	3.43	3.42	3.75	3.23	Median	2.74	2.05
			N4H5S2F1	IQR	2.52	IQR	2.78	2.30	2.54	2.67	2.69	2.51	2.81	3.04	2.23	2.67	2.19	2.67	2.21	2.84	IQR	2.40	1.64
			m/z		3.81		4.22	3.97	3.92	3.48	3.87	3.88	3.71	3.87	3.95	3.54	3.70	3.75	4.46	3.77		3.04	2.98
			2966.4	$\chi^2(14)$	20.840	Z	-	-	-	-	-	-	-	-	-	-	-	-	-	-	Z	-	-1.483
				p value	0.106	p value	-	-	-	-	-	-	-	-	-	-	-	-	-	-	p value	-	0.138
30	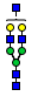	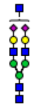		Median	0.07	Median	0.08	0.06	0.07	0.08	0.07	0.07	0.08	0.09	0.06	0.07	0.07	0.08	0.10	0.08	Median	0.05	0.04
			N5H5S2	IQR	0.05	IQR	0.04	0.03	0.04	0.04	0.02	0.05	0.06	0.05	0.03	0.05	0.05	0.05	0.05	0.05	IQR	0.05	0.02
			m/z		0.11		0.12	0.09	0.12	0.12	0.09	0.18	0.13	0.11	0.10	0.11	0.14	0.13	0.11	0.12		0.08	0.08
			3037.4	$\chi^2(14)$	17.930	Z	-	-	-	-	-	-	-	-	-	-	-	-	-	-	Z	-	-0.674
				p value	0.210	p value	-	-	-	-	-	-	-	-	-	-	-	-	-	-	p value	-	0.500
31	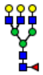	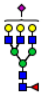		Median	0.09	Median	0.10	0.08	0.09	0.10	0.09	0.08	0.10	0.12	0.08	0.10	0.08	0.09	0.06	0.09	Median	0.07	0.03
			N5H6S1F1	IQR	0.06	IQR	0.05	0.05	0.05	0.07	0.07	0.04	0.06	0.06	0.04	0.06	0.05	0.07	0.04	0.05	IQR	0.03	0.02
			m/z		0.12		0.15	0.11	0.12	0.14	0.11	0.09	0.13	0.15	0.14	0.12	0.12	0.12	0.11	0.15		0.11	0.08
			3054.4	$\chi^2(14)$	12.000	Z	-	-	-	-	-	-	-	-	-	-	-	-	-	-	Z	-	-0.674
				p value	0.606	p value	-	-	-	-	-	-	-	-	-	-	-	-	-	-	p value	-	0.500
32	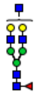	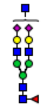		Median	0.66	Median	0.89	0.63	0.66	0.71	0.72	0.65	0.66	0.79	0.60	0.65	0.72	0.70	0.65	0.62	Median	0.59	0.43
			N5H5S2F1	IQR	0.52	IQR	0.48	0.48	0.56	0.57	0.43	0.52	0.56	0.69	0.41	0.56	0.42	0.58	0.45	0.51	IQR	0.51	0.25
			m/z		0.88		1.15	0.85	0.96	0.87	0.89	0.88	0.98	1.09	0.75	0.73	1.03	1.00	0.88	1.13		0.68	0.65
			3211.5	$\chi^2(14)$	12.960	Z	-	-	-	-	-	-	-	-	-	-	-	-	-	-	Z	-	-1.483
				p value	0.530	p value	-	-	-	-	-	-	-	-	-	-	-	-	-	-	p value	-	0.138
33	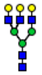	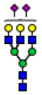		Median	0.66	Median	0.65	0.72	0.82	0.71	0.61	0.60	0.79	0.77	0.57	0.63	0.61	0.66	0.52	0.54	Median	0.62	0.31
			N5H6S2	IQR	0.44	IQR	0.43	0.47	0.55	0.50	0.47	0.43	0.48	0.55	0.41	0.54	0.47	0.44	0.35	0.37	IQR	0.43	0.16
			m/z		0.97		1.11	1.05	1.11	1.02	0.80	1.08	0.97	1.13	1.14	1.05	1.16	1.02	0.81	0.91		0.70	0.36
			3241.5	$\chi^2(14)$	23.880	Z	-	-0.357	-1.580	-0.153	-1.274	-0.866	-0.357	-0.866	-0.357	-	-0.357	-1.886	-0.663	-1.682	Z	-	-2.023
				p value	**0.047**	p value	-	0.721	0.114	0.878	0.203	0.386	0.721	0.386	0.721	-	0.721	0.059	0.508	0.093	p value	-	0.043
34	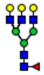	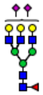		Median	0.10	Median	0.10	0.09	0.11	0.10	0.08	0.11	0.11	0.13	0.11	0.10	0.08	0.13	0.10	0.08	Median	0.08	0.05
			N5H6S2F1	IQR	0.06	IQR	0.07	0.05	0.05	0.07	0.06	0.07	0.08	0.08	0.05	0.07	0.05	0.07	0.04	0.06	IQR	0.06	0.03
			m/z		0.16		0.18	0.16	0.22	0.18	0.19	0.16	0.15	0.16	0.17	0.18	0.24	0.16	0.14	0.12		0.11	0.06
			3415.6	$\chi^2(14)$	13.010	Z	-	-	-	-	-	-	-	-	-	-	-	-	-	-	Z	-	-2.023
				p value	0.526	p value	-	-	-	-	-	-	-	-	-	-	-	-	-	-	p value	-	0.043

Structure				Friedman's test[a]		Wilcoxon's test[b]															Wilcoxon's test[c]		
Peak	Desialylated CFG notation	CFG notation			All conditions		1 Serum ref.	2 6h RT	3 2h RT	4 2h 4°C	5 6h after	6 gel 6h after	7 2 months -80°C	8 2 months -20°C	9 24h 4°C	11 Plasma ref. n=10	12 Vacuum	13 EDTA	14 Heparin	15 Hemolysis water		11 Plasma ref. n=5	16 Hemolysis combined
35	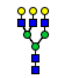	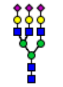	**N5H6S3**	**Median**	5.47	**Median**	6.18	6.22	7.89	5.35	5.03	5.61	4.80	5.41	4.16	5.97	5.93	6.46	6.37	5.99	**Median**	6.34	3.84
			m/z	**IQR**	3.79	**IQR**	3.50	4.15	4.11	4.32	3.67	3.40	4.14	3.13	2.52	4.30	4.35	4.39	3.08	3.44	**IQR**	3.86	1.99
			3602.7		8.79		9.85	11.08	10.21	8.27	6.61	9.28	7.02	8.04	9.02	11.04	10.61	8.88	9.54	8.65		9.61	5.92
				**$\chi^2(14)$**	20.570	**Z**	-	-	-	-	-	-	-	-	-	-	-	-	-	-	**Z**	-	-2.023
				**p value**	0.113	**p value**	-	-	-	-	-	-	-	-	-	-	-	-	-	-	**p value**	-	0.043
36	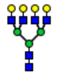	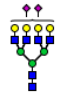	**N6H7S2**	**Median**	0.06	**Median**	0.07	0.06	0.08	0.06	0.06	0.06	0.07	0.08	0.05	0.08	0.07	0.07	0.06	0.07	**Median**	0.07	0.05
			m/z	**IQR**	0.04	**IQR**	0.04	0.04	0.04	0.04	0.04	0.02	0.05	0.06	0.03	0.06	0.06	0.04	0.05	0.03	**IQR**	0.05	0.02
			3690.7		0.09		0.11	0.10	0.10	0.09	0.08	0.09	0.11	0.10	0.09	0.08	0.09	0.09	0.07	0.09		0.10	0.06
				**$\chi^2(14)$**	10.050	**Z**	-	-	-	-	-	-	-	-	-	-	-	-	-	-	**Z**	-	-2.023
				**p value**	0.759	**p value**	-	-	-	-	-	-	-	-	-	-	-	-	-	-	**p value**	-	0.043
37	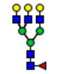	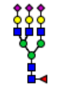	**N5H6S3F1**	**Median**	1.03	**Median**	1.13	1.09	1.30	1.12	0.89	1.06	0.88	1.07	0.85	1.30	1.13	1.13	1.10	0.95	**Median**	1.27	0.57
			m/z	**IQR**	0.61	**IQR**	0.53	0.51	0.64	0.62	0.33	0.66	0.42	0.64	0.60	0.57	0.66	0.88	0.51	0.59	**IQR**	0.53	0.27
			3776.8		1.51		2.06	1.43	1.81	1.63	1.86	1.64	1.45	1.50	1.20	2.43	1.60	1.52	1.53	1.57		2.29	1.25
				**$\chi^2(14)$**	15.830	**Z**	-	-	-	-	-	-	-	-	-	-	-	-	-	-	**Z**	-	-2.023
				**p value**	0.324	**p value**	-	-	-	-	-	-	-	-	-	-	-	-	-	-	**p value**	-	0.043
38	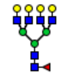	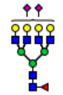	**N6H7S2F1**	**Median**	0.02	**Median**	0.02	0.02	0.01	0.02	0.02	0.01	0.03	0.03	0.02	0.02	0.02	0.02	0.02	0.02	**Median**	0.02	0.02
			m/z	**IQR**	0.01	**IQR**	0.01	0.02	0.01	0.01	0.01	0.01	0.01	0.02	0.01	0.01	0.02	0.01	0.01	0.01	**IQR**	0.01	0.01
			3864.8		0.03		0.04	0.03	0.03	0.03	0.03	0.03	0.05	0.03	0.03	0.04	0.04	0.02	0.03	0.03		0.03	0.03
				**$\chi^2(14)$**	10.540	**Z**	-	-	-	-	-	-	-	-	-	-	-	-	-	-	**Z**	-	-0.405
				**p value**	0.722	**p value**	-	-	-	-	-	-	-	-	-	-	-	-	-	-	**p value**	-	0.686
39	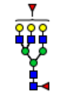	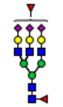	**N5H6S3F2**	**Median**	0.02	**Median**	0.02	0.03	0.01	0.02	0.02	0.02	0.02	0.02	0.02	0.01	0.02	0.02	0.03	0.02	**Median**	0.01	0.01
			m/z	**IQR**	0.01	**IQR**	0.01	0.01	0.01	0.02	0.01	0.01	0.01	0.02	0.01	0.01	0.01	0.01	0.01	0.01	**IQR**	0.01	0.01
			3950.9		0.03		0.04	0.04	0.03	0.04	0.05	0.03	0.04	0.03	0.03	0.03	0.03	0.04	0.04	0.02		0.02	0.03
				**$\chi^2(14)$**	10.740	**Z**	-	-	-	-	-	-	-	-	-	-	-	-	-	-	**Z**	-	-0.135
				**p value**	0.706	**p value**	-	-	-	-	-	-	-	-	-	-	-	-	-	-	**p value**	-	0.893
40	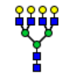	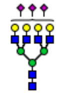	**N6H7S3**	**Median**	0.12	**Median**	0.11	0.15	0.14	0.15	0.10	0.12	0.14	0.12	0.08	0.19	0.17	0.12	0.10	0.14	**Median**	0.15	0.06
			m/z	**IQR**	0.09	**IQR**	0.08	0.08	0.10	0.10	0.07	0.09	0.09	0.09	0.05	0.12	0.10	0.11	0.07	0.08	**IQR**	0.11	0.05
			4052.0		0.17		0.24	0.18	0.23	0.16	0.15	0.19	0.18	0.16	0.19	0.22	0.23	0.16	0.15	0.19		0.20	0.12
				**$\chi^2(14)$**	16.490	**Z**	-	-	-	-	-	-	-	-	-	-	-	-	-	-	**Z**	-	-2.023
				**p value**	0.284	**p value**	-	-	-	-	-	-	-	-	-	-	-	-	-	-	**p value**	-	0.043
41	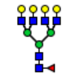	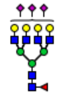	**N6H7S3F1**	**Median**	0.03	**Median**	0.02	0.03	0.03	0.04	0.03	0.04	0.03	0.04	0.03	0.03	0.03	0.03	0.03	0.02	**Median**	0.03	0.02
			m/z	**IQR**	0.02	**IQR**	0.01	0.02	0.01	0.02	0.02	0.02	0.02	0.02	0.02	0.02	0.02	0.02	0.02	0.02	**IQR**	0.01	0.01
			4226.1		0.05		0.05	0.05	0.05	0.06	0.05	0.05	0.04	0.06	0.04	0.07	0.05	0.04	0.06	0.04		0.06	0.04
				**$\chi^2(14)$**	13.600	**Z**	-	-	-	-	-	-	-	-	-	-	-	-	-	-	**Z**	-	-0.944
				**p value**	0.480	**p value**	-	-	-	-	-	-	-	-	-	-	-	-	-	-	**p value**	-	0.345
42	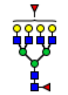	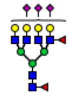	**N6H7S3F2**	**Median**	0.01	**Median**	0.02	0.01	0.01	0.02	0.01	0.01	0.02	0.01	0.01	0.02	0.01	0.01	0.01	0.01	**Median**	0.02	0.01
			m/z	**IQR**	0.01	**IQR**	0.01	0.01	0.01	0.01	0.01	0.01	0.01	0.00	0.01	0.01	0.01	0.01	0.01	0.01	**IQR**	0.01	0.00
			4400.2		0.02		0.03	0.02	0.03	0.03	0.02	0.02	0.03	0.02	0.02	0.02	0.02	0.02	0.02	0.01		0.02	0.02
				**$\chi^2(14)$**	13.690	**Z**	-	-	-	-	-	-	-	-	-	-	-	-	-	-	**Z**	-	-2.023
				**p value**	0.473	**p value**	-	-	-	-	-	-	-	-	-	-	-	-	-	-	**p value**	-	0.043
43	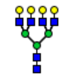	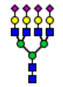	**N6H7S4**	**Median**	0.23	**Median**	0.19	0.24	0.27	0.22	0.17	0.23	0.18	0.17	0.10	0.49	0.37	0.31	0.27	0.35	**Median**	0.53	0.17
			m/z	**IQR**	0.14	**IQR**	0.08	0.12	0.15	0.17	0.10	0.14	0.11	0.11	0.06	0.27	0.25	0.22	0.16	0.18	**IQR**	0.26	0.13
			4413.2		0.35		0.46	0.36	0.47	0.32	0.25	0.32	0.27	0.26	0.39	0.58	0.53	0.36	0.36	0.47		0.60	0.30
				**$\chi^2(14)$**	32.400	**Z**	-	-0.051	-0.459	-0.255	-0.561	-0.357	-0.968	-0.968	-0.663	-	-1.070	-2.090	-1.886	-1.274	**Z**	-	-1.753
				**p value**	**0.004**	**p value**	-	0.959	0.646	0.799	0.575	0.721	0.333	0.333	0.508	-	0.285	0.037	0.059	0.203	**p value**	-	0.080

Structure				Friedman's test[a]		Wilcoxon's test[b]															Wilcoxon's test[c]		
Peak	Desialylated CFG notation	CFG notation			All conditions		1 Serum ref.	2 6h RT	3 2h RT	4 2h 4°C	5 6h after	6 gel 6h after	7 2 months -80°C	8 2 months -20°C	9 24h 4°C	11 Plasma ref. n=10	12 Vacuum	13 EDTA	14 Heparin	15 Hemolysis water		11 Plasma ref. n=5	16 Hemolysis combined
				**Median**	0.05	**Median**	0.04	0.04	0.05	0.06	0.06	0.05	0.04	0.05	0.04	0.07	0.07	0.07	0.06	0.07	**Median**	0.09	0.05
	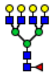	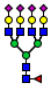	**N6H7S4F1**	**IQR**	0.03	**IQR**	0.02	0.03	0.04	0.03	0.03	0.03	0.02	0.03	0.03	0.04	0.05	0.03	0.04	0.03	**IQR**	0.03	0.02
44			m/z		0.08		0.11	0.06	0.07	0.11	0.08	0.08	0.06	0.08	0.05	0.16	0.11	0.08	0.13	0.10		0.17	0.08
			4587.4	**χ^2(14)**	24.480	**Z**	-	-0.561	-0.459	-1.070	-0.459	-0.357	-0.153	-0.051	-0.968	-	-0.561	-0.255	-1.784	-1.478	**Z**	-	-1.753
				**p value**	**0.040**	**p value**	-	0.575	0.646	0.285	0.646	0.721	0.878	0.959	0.333	-	0.575	0.799	0.074	0.139	**p value**	-	0.080
				**Median**	0.02	**Median**	0.02	0.01	0.01	0.02	0.02	0.01	0.01	0.02	0.02	0.01	0.01	0.02	0.03	0.01	**Median**	0.01	0.02
	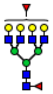	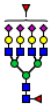	**N6H7S4F2**	**IQR**	0.01	**IQR**	0.01	0.01	0.00	0.01	0.01	0.01	0.01	0.01	0.01	0.01	0.01	0.01	0.02	0.01	**IQR**	0.01	0.01
45			m/z		0.03		0.03	0.02	0.03	0.03	0.03	0.02	0.02	0.03	0.03	0.03	0.03	0.02	0.04	0.03		0.03	0.03
			4761.5	**χ^2(14)**	17.110	**Z**	-	-	-	-	-	-	-	-	-	-	-	-	-	-	**Z**	-	-0.674
				**p value**	0.250	**p value**	-	-	-	-	-	-	-	-	-	-	-	-	-	-	**p value**	-	0.500
				**Median**	0.01	**Median**	0.01	0.01	0.01	0.02	0.01	0.00	0.01	0.01	0.01	0.01	0.01	0.01	0.01	0.01	**Median**	0.01	0.01
	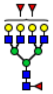	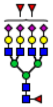	**N6H7S4F3**	**IQR**	0.01	**IQR**	0.01	0.00	0.00	0.01	0.01	0.00	0.00	0.00	0.01	0.01	0.00	0.01	0.01	0.01	**IQR**	0.01	0.01
46			m/z		0.02		0.02	0.02	0.01	0.03	0.01	0.01	0.03	0.02	0.02	0.02	0.02	0.01	0.02	0.01		0.01	0.01
			4935.7	**χ^2(14)**	17.030	**Z**	-	-	-	-	-	-	-	-	-	-	-	-	-	-	**Z**	-	-0.674
				**p value**	0.255	**p value**	-	-	-	-	-	-	-	-	-	-	-	-	-	-	**p value**	-	0.500
