# Supplementary material for: The effect of blood sampling and preanalytical processing on human N-glycome
Source: PLoS One. 2018 Jul 11;13(7):e0200507. doi: 10.1371/journal.pone.0200507 (PMC6040761; doi:10.1371/journal.pone.0200507)
Supplement: S4 Table — Relative intensities of individual N-glycans (median, IQR) were tested for differences between all groups (n = 15) (a) by Friedman’s test and p-values < .05 were considered statistically significant (marked red). Only the structures, which were detected as significantly different by Friedman’s test, were further analysed by a Wilcoxon's post-hoc test to identify if such differences occurred as a result of deviation from the standard procedure. (b) Wilcoxon's test was used to compare standard conditions with the altered conditions for plasma and serum separately. Statistical tests between two non-standard conditions were not performed, since these are not relevant for this study. Nine tests were performed for serum conditions, resulting in a Bonferroni corrected p value of .0056. For plasma conditions five tests were performed including (c) Wilcoxon's test where results from only 5 individuals were compared, therefore the Bonferroni corrected p value for plasma samples was .01. The Oxford notations are: pentasaccharide core (A0) consists of three mannose residues and two N-acetylglucosamines (GlcNAc); F core fucose; aF antennary fucose; Ax, number GlcNAc attached to the core; B, bisecting GlcNAc; Gx, number of β1–4 linked galactose (G) on antennae; [3]G1 and [6]G1 indicates that the galactose is on the antenna of the α1–3 or α1–6 mannose. The [x] indicate the linkage type: in A2[3]G1 the galactose is linked to the α1–3 mannose, and in A3[2,2,6] the N-acetylglucosamines are β1-2-, β1-2-, and β1-6-linked to the trimannosyl core. In A3G3[3], the [3] indicates a β1–3 linkage between terminal G and GlcNAc. (PDF) [file pone.0200507.s007.pdf]

S4 Table. Results of CE-LIF measurements and statistical analysis. Relative intensities of individual N-glycans (median, IQR) were tested for differences between all groups (n=15) (a) by Friedman's test and p < .05 were considered statistically significant. Only the structures, which were detected as significantly different by Friedman's test, were further analysed by a Wilcoxon's post-hoc test to identify if such differences occurred as a result of deviation from the standard procedure. (b) Wilcoxon's test was used to compare standard conditions with the altered conditions for plasma and serum separately. Statistical tests between two non-standard conditions were not performed, since these are not relevant for this study. Nine tests were performed for serum conditions, resulting in a Bonferroni corrected p value of .0056. For plasma conditions five tests were performed including (c) Wilcoxon's test where results from only 5 individuals were compared, therefore the Bonferroni corrected p value for plasma samples was .01. The Oxford notaions are: pentasaccharide core (A0) consists of three mannose residues and two N-acetylglucosamines (GlcNAc); F core fucose; aF antennary fucose; Ax, number GlcNAc attached to the core; B, bisecting GlcNAc; Gx, number of β1-4 linked galactose (G) on antennae; [3]G1 and [6]G1 indicates that the galactose is on the antenna of the α1-3 or α1-6 mannose. The [x] indicate the linkage type: in A2[3]G1 the galactose is linked to the α1-3 mannose, and in A3[2,2,6] the N-acetylglucosamines are β1-2-, β1-2-, and β1-6-linked to the trimannosyl core. In A3G3[3], the [3] indicates a β1-3 linkage between terminal G and GlcNAc.

Peak	GU	Structure			Friedman's test[a]		Wilcoxon's test[b]																Wilcoxon's test[c]		
		Desialylated CFG notation	Oxford notation	Oxford notation name		All		1 Serum ref.	2 6h RT	3 2h RT	4 2h 4°C	5 6h after	6 gel 6h after	7 2M -80°C	8 2M -20°C	9 24h 4°C	10 48h 4°C	11 Plasma ref. n=10	12 Vacuum	13 EDTA	14 Heparin	15 Hemolysis water		11 Plasma ref. n=5	16 Hemolysis combined
1	4.93	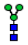	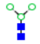	M3	Median	0.11	Median	0.09	0.11	0.11	0.10	0.11	0.10	0.10	0.11	0.10	0.10	0.12	0.12	0.09	0.39	0.12	Median	0.12	0.13
					IQR	0.07	IQR	0.07	0.08	0.07	0.03	0.04	0.03	0.05	0.05	0.06	0.04	0.08	0.07	0.06	0.21	0.11	IQR	0.09	0.09
						0.13		0.13	0.14	0.12	0.11	0.13	0.11	0.12	0.12	0.12	0.12	0.16	0.14	0.12	0.52	0.21		0.13	0.16
					χ^2(14)	34.331	Z	-	-0.866	-0.051	-0.561	-0.866	-1.274	-0.866	-0.459	-0.153	-0.357	-	-0.866	-1.682	-2.599	-0.663	Z	-	-1.214
					p value	**0.002**	p value	-	0.386	0.959	0.575	0.386	0.203	0.386	0.646	0.878	0.721	-	0.386	0.093	**0.009**	0.508	p value	-	0.225
2	5.77	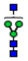	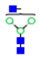	A1	Median	0.13	Median	0.11	0.13	0.12	0.13	0.11	0.11	0.13	0.13	0.15	0.11	0.13	0.13	0.13	0.12	0.13	Median	0.13	0.14
					IQR	0.10	IQR	0.09	0.09	0.11	0.10	0.09	0.09	0.11	0.10	0.08	0.08	0.09	0.11	0.08	0.10	0.10	IQR	0.11	0.11
						0.24		0.26	0.31	0.24	0.28	0.24	0.27	0.32	0.28	0.27	0.25	0.27	0.27	0.28	0.26	0.32		0.31	0.34
					χ^2(14)	29.730	Z	-	-1.070	-0.561	-1.274	-2.090	-0.459	-1.988	-1.478	-0.663	-1.478	-	-1.274	-0.968	-0.051	-1.682	Z	-	-1.214
					p value	**0.008**	p value	-	0.285	0.575	0.203	0.037	0.646	0.047	0.139	0.508	0.139	-	0.203	0.333	0.959	0.093	p value	-	0.225
3	6.72	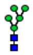	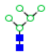	M5	Median	0.97	Median	0.98	0.93	0.95	0.96	0.99	1.01	0.97	0.95	0.98	0.95	0.95	1.01	0.96	0.89	0.99	Median	0.92	1.00
					IQR	0.76	IQR	0.77	0.78	0.83	0.70	0.74	0.75	0.69	0.68	0.91	0.79	0.72	0.82	0.85	0.75	0.71	IQR	0.65	0.75
						1.10		1.14	1.17	1.10	1.10	1.18	1.14	1.09	1.02	1.08	1.27	1.06	1.24	1.10	1.18	1.18		1.19	1.33
					χ^2(14)	23.210	Z	-	-	-	-	-	-	-	-	-	-	-	-	-	-	-	Z	-	-2.023
					p value	0.057	p value	-	-	-	-	-	-	-	-	-	-	-	-	-	-	-	p value	-	0.043
4	7.17	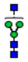	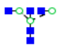	A2B	Median	0.52	Median	0.46	0.49	0.52	0.49	0.48	0.51	0.54	0.54	0.52	0.58	0.54	0.62	0.56	0.55	0.54	Median	0.54	0.54
					IQR	0.45	IQR	0.39	0.41	0.46	0.22	0.34	0.38	0.47	0.49	0.36	0.47	0.50	0.48	0.46	0.35	0.31	IQR	0.42	0.52
						0.60		0.54	0.58	0.57	0.60	0.57	0.57	0.63	0.59	0.59	0.64	0.63	0.75	0.58	0.61	0.61		0.61	0.70
					χ^2(14)	19.430	Z	-	-	-	-	-	-	-	-	-	-	-	-	-	-	-	Z	-	-0.674
					p value	0.149	p value	-	-	-	-	-	-	-	-	-	-	-	-	-	-	-	p value	-	0.500
5	7.62<br>7.74<br>7.74	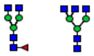	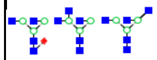	FA2<br>A3<br>A3	Median	2.61	Median	2.42	2.70	2.60	2.67	2.81	2.58	2.51	2.25	2.60	2.63	2.70	2.63	2.47	2.54	2.76	Median	2.78	3.01
					IQR	2.10	IQR	1.92	2.13	2.08	2.11	2.13	1.91	2.08	1.72	2.01	1.97	2.10	2.47	2.12	2.04	2.24	IQR	2.42	2.28
						2.99		3.16	3.00	2.96	3.03	3.06	3.00	2.95	2.77	3.06	3.08	3.09	3.01	3.02	2.82	3.12		3.42	3.37
					χ^2(14)	54.827	Z	-	-1.172	-0.764	-0.968	-1.784	-0.415	-0.968	-1.478	-1.478	-0.357	-	-0.968	-1.988	-2.701	-1.376	Z	-	-0.135
					p value	**< 0.0001**	p value	-	0.241	0.445	0.333	0.074	0.678	0.333	0.139	0.139	0.721	-	0.333	0.047	**0.007**	0.169	p value	-	0.893
6	7.95	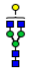	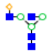	A2[3]G1	Median	0.54	Median	0.53	0.54	0.52	0.54	0.54	0.53	0.54	0.54	0.51	0.52	0.55	0.53	0.56	0.50	0.58	Median	0.54	0.59
					IQR	0.49	IQR	0.48	0.50	0.42	0.51	0.49	0.45	0.50	0.51	0.46	0.45	0.49	0.48	0.50	0.42	0.53	IQR	0.47	0.49
						0.58		0.58	0.55	0.55	0.59	0.57	0.56	0.58	0.60	0.56	0.57	0.60	0.61	0.59	0.54	0.61		0.58	0.62
					χ^2(14)	43.170	Z	-	-0.968	-1.580	-0.255	-0.357	-0.357	-0.153	-0.357	-1.784	-0.663	-	-1.580	-0.561	-2.803	-1.478	Z	-	-1.753
					p value	**< 0.0001**	p value	-	0.333	0.114	0.799	0.721	0.721	0.878	0.721	0.074	0.508	-	0.114	0.575	**0.005**	0.139	p value	-	0.080
7	8.07	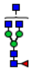	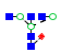	FA2B	Median	0.61	Median	0.61	0.61	0.59	0.60	0.62	0.62	0.63	0.62	0.61	0.61	0.60	0.61	0.62	0.56	0.64	Median	0.58	0.62
					IQR	0.57	IQR	0.58	0.55	0.57	0.55	0.58	0.58	0.60	0.57	0.58	0.60	0.55	0.56	0.55	0.53	0.59	IQR	0.55	0.59
						0.69		0.67	0.68	0.67	0.71	0.70	0.71	0.71	0.70	0.70	0.71	0.71	0.70	0.69	0.64	0.71		0.72	0.72
					χ^2(14)	47.410	Z	-	-0.663	-1.784	-0.459	-1.682	-0.357	-0.866	-0.153	-0.255	-1.172	-	-0.561	-1.172	-2.701	-1.478	Z	-	-1.753
					p value	**< 0.0001**	p value	-	0.508	0.074	0.646	0.093	0.721	0.386	0.878	0.799	0.241	-	0.575	0.241	**0.007**	0.139	p value	-	0.080
8	8.45	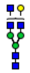	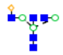	A2B[3]G1	Median	0.56	Median	0.56	0.54	0.53	0.55	0.57	0.59	0.57	0.56	0.55	0.57	0.54	0.54	0.54	0.52	0.59	Median	0.56	0.61
					IQR	0.52	IQR	0.48	0.52	0.48	0.51	0.54	0.52	0.51	0.51	0.53	0.53	0.49	0.50	0.51	0.47	0.54	IQR	0.48	0.55
						0.60		0.58	0.59	0.59	0.62	0.60	0.63	0.69	0.64	0.63	0.66	0.61	0.65	0.59	0.57	0.62		0.68	0.76
					χ^2(14)	48.600	Z	-	-0.255	-1.172	-0.153	-1.682	-1.172	-0.968	-0.357	-1.274	-1.682	-	-0.051	-0.561	-2.090	-2.701	Z	-	-2.023
					p value	**< 0.0001**	p value	-	0.799	0.241	0.878	0.093	0.241	0.333	0.721	0.203	0.093	-	0.959	0.575	0.037	**0.007**	p value	-	0.043

Peak	GU	Structure			Friedman's test[a]		Wilcoxon's test[b]																Wilcoxon's test[c]		
		Desialylated CFG notation	Oxford notation	Oxford notation name		All		1 Serum ref.	2 6h RT	3 2h RT	4 2h 4°C	5 6h after	6 gel 6h after	7 2M -80°C	8 2M -20°C	9 24h 4°C	10 48h 4°C	11 Plasma ref. n=10	12 Vacuum	13 EDTA	14 Heparin	15 Hemolysis water		11 Plasma ref. n=5	16 Hemolysis combined
9	8.58	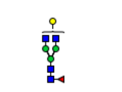	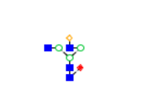	FA2[6]G1	Median	1.83	Median	1.80	1.83	1.88	1.84	1.77	1.81	1.83	1.66	1.86	1.82	1.73	1.85	1.79	1.83	1.70	Median	2.14	1.92
					IQR	1.65	IQR	1.62	1.71	1.72	1.73	1.68	1.68	1.60	1.46	1.64	1.66	1.42	1.66	1.68	1.59	1.45	IQR	2.00	1.85
						2.13		2.19	2.24	2.29	2.21	2.02	2.26	2.15	1.96	2.25	2.22	2.20	2.10	2.02	2.32	2.24		2.74	2.78
					$\chi^2(14)$	49.190	Z	-	-0.357	-0.968	-0.153	-0.968	-0.764	-1.070	-2.293	-0.866	-0.153	-	-0.968	-0.153	-1.376	-0.357	Z	-	-0.944
					p value	< 0.0001	p value	-	0.721	0.333	0.878	0.333	0.445	0.285	0.022	0.386	0.878	-	0.333	0.878	0.169	0.721	p value	-	0.345
10	8.88	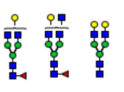	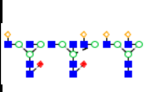	FA2[3]G1	Median	51.18	Median	52.13	50.01	50.67	51.83	52.48	51.22	49.93	51.01	50.85	50.92	51.60	50.75	52.84	51.34	51.86	Median	50.89	50.17
	8.91			FA2B[6]G1	IQR	49.41	IQR	49.69	48.81	48.41	49.37	50.56	49.33	48.41	49.02	49.17	48.85	50.52	48.82	49.79	49.43	50.00	IQR	49.26	49.46
						52.88		53.95	52.38	52.05	53.12	54.30	53.23	51.82	52.26	52.57	53.60	53.46	52.50	54.28	52.66	53.89		51.75	51.60
					$\chi^2(14)$	38.220	Z	-	-1.682	-1.784	-0.663	-0.764	-0.663	-2.395	-1.478	-0.866	-1.070	-	-1.682	-1.274	-1.784	-0.255	Z	-	-0.674
	8.93			A2G2	p value	< 0.0001	p value	-	0.093	0.074	0.508	0.445	0.508	0.017	0.139	0.386	0.285	-	0.093	0.203	0.074	0.799	p value	-	0.500
11	9.23	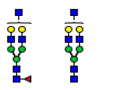	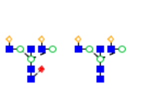	FA2B[3]G1	Median	1.71	Median	1.59	1.71	1.70	1.76	1.77	1.86	1.85	1.66	1.82	1.73	1.56	1.61	1.74	1.54	1.69	Median	1.64	1.78
					IQR	1.36	IQR	0.89	1.05	1.16	1.04	1.09	1.34	1.01	0.66	0.49	1.23	1.03	1.44	1.45	0.62	1.20	IQR	1.43	1.59
						1.92		1.94	1.85	1.91	1.93	1.94	2.08	1.98	2.02	1.93	2.14	1.90	1.97	1.93	1.78	1.96		1.79	2.04
	9.41			A2BG2	$\chi^2(14)$	24.905	Z	-	-0.280	-0.255	-0.140	-0.840	-1.362	-1.260	-0.296	.000d	-1.599	-	-0.296	-1.599	-0.560	-2.073	Z	-	-2.023
					p value	0.036	p value	-	0.779	0.799	0.889	0.401	0.173	0.208	0.767	1.000	0.110	-	0.767	0.110	0.575	0.038	p value	-	0.043
12	9.69	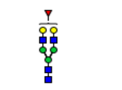	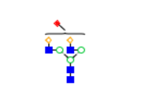	aFA2G2	Median	1.08	Median	0.90	1.06	1.13	0.87	1.08	1.00	1.28	1.25	1.09	1.19	1.14	1.25	1.02	1.16	1.12	Median	1.10	1.00
					IQR	0.87	IQR	0.74	0.92	1.00	0.71	0.85	0.73	1.06	0.99	0.77	0.83	1.04	0.74	0.73	0.72	0.95	IQR	0.87	0.91
						1.43		1.12	1.49	1.48	1.02	1.30	1.47	1.50	1.41	1.39	1.62	1.47	1.53	1.42	1.67	1.43		1.35	1.33
					$\chi^2(14)$	22.540	Z	-	-	-	-	-	-	-	-	-	-	-	-	-	-	-	Z	-	-0.405
					p value	0.680	p value	-	-	-	-	-	-	-	-	-	-	-	-	-	-	-	p value	-	0.686
13	9.82	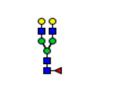	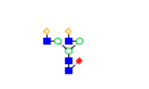	FA2G2	Median	13.69	Median	13.63	13.51	13.71	13.81	13.84	14.04	13.94	13.79	13.77	14.09	13.37	13.69	13.66	13.69	13.65	Median	13.12	13.08
					IQR	13.07	IQR	13.07	13.00	12.82	13.01	13.30	13.01	13.10	13.20	13.53	13.55	12.81	12.59	12.79	12.71	12.98	IQR	12.60	12.79
						14.41		14.57	14.61	14.74	14.47	14.84	14.51	14.72	14.69	14.58	14.76	13.99	14.10	14.43	14.17	14.11		14.03	14.20
					$\chi^2(14)$	38.050	Z	-	-0.153	-0.051	-0.561	-1.784	-0.663	-1.172	-1.274	-1.376	-1.580	-	-0.051	-1.274	-1.070	-2.497	Z	-	-0.674
					p value	0.001	p value	-	0.878	0.959	0.575	0.074	0.508	0.241	0.203	0.169	0.114	-	0.959	0.203	0.285	0.013	p value	-	0.500
14	10.14	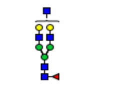	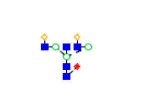	FA2BG2	Median	4.31	Median	3.93	4.12	4.16	4.17	4.27	4.46	4.54	4.50	4.29	4.58	4.07	4.52	4.35	4.10	4.59	Median	4.57	4.88
					IQR	3.87	IQR	3.76	3.68	3.60	3.42	4.06	3.66	4.06	3.92	4.07	3.83	3.63	3.86	3.99	3.45	3.91	IQR	3.87	4.18
						5.03		4.81	4.90	4.61	5.33	4.93	5.31	5.62	5.32	4.77	5.24	4.79	5.31	5.18	5.08	5.05		5.45	5.65
					$\chi^2(14)$	36.240	Z	-	-0.459	-0.255	-0.459	-2.599	-1.172	-2.803	-2.293	-1.886	-1.886	-	-1.784	-0.255	-1.478	-0.153	Z	-	-2.023
					p value	0.001	p value	-	0.646	0.799	0.646	0.009	0.241	0.005	0.022	0.059	0.059	-	0.074	0.799	0.139	0.878	p value	-	0.043
15	10.95	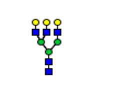	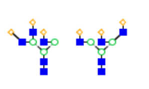	A3G3[3]	Median	1.55	Median	1.53	1.57	1.55	1.58	1.53	1.58	1.54	1.49	1.57	1.59	1.42	1.56	1.51	1.53	1.53	Median	1.56	1.53
					IQR	1.43	IQR	1.40	1.46	1.47	1.45	1.44	1.45	1.35	1.40	1.51	1.36	1.31	1.45	1.36	1.40	1.39	IQR	1.40	1.42
						1.67		1.72	1.68	1.71	1.71	1.74	1.73	1.65	1.65	1.74	1.72	1.57	1.68	1.66	1.70	1.62		1.69	1.70
				A3[6]G3	$\chi^2(14)$	54.980	Z	-	-1.376	-1.682	-1.988	-1.172	-2.803	-1.172	-1.070	-2.497	-1.070	-	-2.395	-2.497	-2.803	-1.376	Z	-	-0.405
					p value	< 0.0001	p value	-	0.169	0.093	0.047	0.241	0.005	0.241	0.285	0.013	0.285	-	0.017	0.013	0.005	0.169	p value	-	0.686
16	11.16	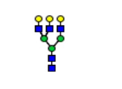	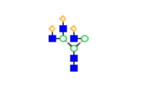	A3G3	Median	12.21	Median	12.70	12.66	12.40	12.33	11.82	12.11	11.88	12.54	12.34	11.74	12.35	11.99	11.74	12.42	11.30	Median	11.89	10.72
					IQR	9.99	IQR	9.73	9.42	9.96	10.18	9.24	9.79	10.68	10.79	9.21	10.20	9.92	10.59	8.56	10.01	9.24	IQR	9.82	9.06
						13.58		13.98	14.45	14.18	14.34	13.71	13.92	14.33	14.52	13.57	13.41	13.73	13.65	13.94	13.57	13.23		13.56	12.86
					$\chi^2(14)$	36.850	Z	-	-0.255	-0.357	-1.172	-1.478	-0.561	-0.459	-1.376	-1.478	-1.070	-	-0.153	-1.682	-1.376	-2.803	Z	-	-2.023
					p value	0.001	p value	-	0.799	0.721	0.241	0.139	0.575	0.646	0.169	0.139	0.285	-	0.878	0.093	0.169	0.005	p value	-	0.043
17	11.80	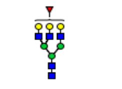	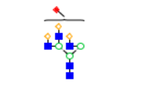	aFA3G3	Median	1.87	Median	2.28	2.00	1.98	1.85	1.60	1.92	1.93	1.95	1.96	1.91	1.71	1.61	1.90	1.82	1.52	Median	1.91	1.69
					IQR	0.99	IQR	0.87	0.86	0.85	1.03	0.39	0.79	1.30	0.92	0.83	0.84	0.77	0.82	0.83	0.77	0.68	IQR	0.88	0.94
						2.79		2.84	3.03	3.19	2.69	2.97	2.91	3.07	3.11	2.98	2.98	2.72	2.18	2.40	2.94	2.15		2.67	2.82
					$\chi^2(14)$	71.270	Z	-	-0.255	-0.153	-1.478	-0.663	-0.255	-0.968	-0.255	-0.357	-1.070	-	-0.764	-0.764	-2.701	-1.988	Z	-	-0.944
					p value	< 0.0001	p value	-	0.799	0.878	0.139	0.508	0.799	0.333	0.799	0.721	0.285	-	0.445	0.445	0.007	0.047	p value	-	0.345
18	12.02	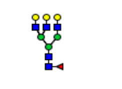	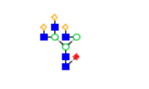	FA3G3	Median	1.21	Median	1.28	1.21	1.24	1.28	1.31	1.22	1.21	1.30	1.30	1.29	1.27	1.26	1.12	1.34	1.16	Median	1.00	0.91
					IQR	0.96	IQR	1.01	0.91	0.98	0.90	0.92	0.94	1.06	1.08	0.91	0.92	0.96	0.98	0.91	0.94	0.97	IQR	0.84	0.76
						1.75		1.71	1.87	1.86	1.60	1.74	1.75	1.98	2.04	1.77	1.66	1.88	1.81	1.82	1.98	1.71		1.67	1.60
					$\chi^2(14)$	26.490	Z	-	-0.255	-0.051	-1.886	-0.561	-0.866	-0.764	-1.784	-0.459	-0.255	-	-0.051	-1.274	-1.784	-1.070	Z	-	-2.023
					p value	0.022	p value	-	0.799	0.959	0.059	0.575	0.386	0.445	0.074	0.646	0.799	-	0.959	0.203	0.074	0.285	p value	-	0.043

Peak	GU	Structure			Friedman's test[a]		Wilcoxon's test[b]																Wilcoxon's test[c]		
		Desialylated CFG notation	Oxford notation	Oxford notation name		All		1 Serum ref.	2 6h RT	3 2h RT	4 2h 4°C	5 6h after	6 gel 6h after	7 2M -80°C	8 2M -20°C	9 24h 4°C	10 48h 4°C	11 Plasma ref. n=10	12 Vacuum	13 EDTA	14 Heparin	15 Hemolysis water		11 Plasma ref. n=5	16 Hemolysis combined
19	12.61	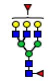	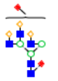	FaFA3G3	Median	0.27	Median	0.27	0.28	0.29	0.27	0.29	0.29	0.29	0.28	0.26	0.26	0.22	0.27	0.24	0.28	0.30	Median	0.25	0.28
					IQR	0.16	IQR	0.20	0.14	0.14	0.21	0.11	0.15	0.17	0.16	0.15	0.15	0.12	0.15	0.14	0.19	0.19	IQR	0.17	0.18
						0.39		0.37	0.42	0.45	0.40	0.39	0.46	0.35	0.37	0.43	0.44	0.33	0.36	0.41	0.49	0.39		0.43	0.44
					χ^2(14)	28.693	Z	-	-1.682	-1.172	-2.073	-0.051	-1.886	-0.051	-0.051	-0.866	-1.070	-	-1.784	-0.459	-2.497	-1.886	Z		-1.214
					p value	**0.011**	p value	-	0.093	0.241	0.038	0.959	0.059	0.959	0.959	0.386	0.285	-	0.074	0.646	0.013	0.059	p value		0.225
20	13.00	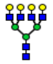	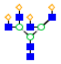	A4G4	Median	2.15	Median	2.08	2.24	2.24	2.17	1.73	2.09	2.17	2.15	1.94	1.98	2.33	2.52	2.03	2.20	2.18	Median	2.79	2.54
					IQR	1.91	IQR	1.83	1.79	1.91	1.94	1.21	1.76	2.02	2.00	1.67	1.66	2.06	2.11	1.65	2.01	2.03	IQR	2.33	2.30
						2.52		2.38	2.74	2.76	2.45	2.07	2.46	2.51	2.30	2.36	2.22	2.82	2.78	2.56	2.78	2.57		3.02	3.03
					χ^2(14)	52.797	Z	-	-0.764	-1.274	-0.561	-2.497	-0.357	-1.274	-0.561	-0.866	-1.478	-	-1.784	-2.191	-0.459	-1.376	Z		-0.674
					p value	**< 0.0001**	p value	-	0.445	0.203	0.575	0.013	0.721	0.203	0.575	0.386	0.139	-	0.074	0.028	0.646	0.169	p value		0.500
21	13.62<br>13.77	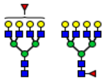	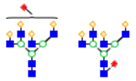	aFA4G4<br>FA4G4	Median	0.38	Median	0.44	0.65	0.66	0.32	0.06	0.24	0.43	0.42	0.51	0.31	0.39	0.18	0.16	0.64	0.18	Median	0.55	0.50
					IQR	0.05	IQR	0.09	0.00	0.07	0.00	0.00	0.00	0.21	0.14	0.09	0.03	0.11	0.04	0.05	0.04	0.00	IQR	0.12	0.22
						0.71		0.59	0.90	0.88	0.74	0.43	0.66	0.72	0.71	0.84	0.56	0.79	1.04	0.55	0.78	0.41		0.78	1.29
					χ^2(14)	22.523	Z	-	-	-	-	-	-	-	-	-	-	-	-	-	-	-	Z		-0.730
					p value	0.068	p value	-	-	-	-	-	-	-	-	-	-	-	-	-	-	-	p value		0.465
22	14.22	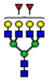	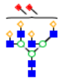	aF2A4G4	Median	0.03	Median	0.03	0.02	0.02	0.03	0.02	0.03	0.00	0.00	0.01	0.01	0.08	0.04	0.02	0.08	0.12	Median	0.12	0.09
					IQR	0.00	IQR	0.01	0.00	0.00	0.00	0.00	0.01	0.00	0.00	0.00	0.00	0.00	0.02	0.00	0.02	0.07	IQR	0.08	0.08
						0.08		0.05	0.06	0.09	0.05	0.07	0.06	0.06	0.04	0.06	0.03	0.14	0.14	0.08	0.11	0.15		0.15	0.13
					χ^2(14)	51.396	Z	-	-0.980	-0.178	-1.120	-1.260	-0.561	-1.955	-2.100	-0.652	-2.100	-	-0.059	-1.352	0.000	-1.988	Z		-0.405
					p value	**< 0.0001**	p value	-	0.327	0.859	0.263	0.208	0.575	0.051	0.036	0.515	0.036	-	0.953	0.176	1.000	0.047	p value		0.686
23	14.69	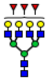	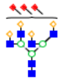	aF3A4G4	Median	0.10	Median	0.11	0.10	0.09	0.10	0.08	0.09	0.07	0.06	0.09	0.09	0.14	0.17	0.09	0.11	0.24	Median	0.24	0.22
					IQR	0.07	IQR	0.08	0.08	0.08	0.04	0.05	0.06	0.05	0.05	0.08	0.06	0.10	0.14	0.05	0.07	0.18	IQR	0.17	0.19
						0.15		0.12	0.13	0.12	0.16	0.12	0.15	0.09	0.08	0.12	0.10	0.24	0.19	0.16	0.14	0.30		0.28	0.23
					χ^2(14)	71.270	Z	-	-0.561	-0.764	-0.153	-0.866	-0.051	-2.497	-2.803	-1.274	-1.682	-	-0.561	-2.395	-2.293	-2.293	Z		-0.674
					p value	**< 0.0001**	p value	-	0.575	0.445	0.878	0.386	0.959	0.013	**0.005**	0.203	0.093	-	0.575	0.017	0.022	0.022	p value		0.500
